# An asynchronous wireless network for capturing event-driven data from large populations of autonomous sensors

**DOI:** 10.1038/s41928-024-01134-y

**Published:** 2024-03-19

**Authors:** Jihun Lee, Ah-Hyoung Lee, Vincent Leung, Farah Laiwalla, Miguel Angel Lopez-Gordo, Lawrence Larson, Arto Nurmikko

**Affiliations:** 1 School of Engineering, Brown University, Providence, RI USA; 2 Electrical and Computer Engineering, Baylor University, Waco, TX USA; 3 Department of Signal Theory, Telematics and Communications, University of Granada, Granada, Spain; 4 Carney Institute for Brain Science, Brown University, Providence, RI USA

**Keywords:** Biomedical engineering, Electrical and electronic engineering

## Abstract

Networks of spatially distributed radiofrequency identification sensors could be used to collect data in wearable or implantable biomedical applications. However, the development of scalable networks remains challenging. Here we report a wireless radiofrequency network approach that can capture sparse event-driven data from large populations of spatially distributed autonomous microsensors. We use a spectrally efficient, low-error-rate asynchronous networking concept based on a code-division multiple-access method. We experimentally demonstrate the network performance of several dozen submillimetre-sized silicon microchips and complement this with large-scale in silico simulations. To test the notion that spike-based wireless communication can be matched with downstream sensor population analysis by neuromorphic computing techniques, we use a spiking neural network machine learning model to decode prerecorded open source data from eight thousand spiking neurons in the primate cortex for accurate prediction of hand movement in a cursor control task.

## Abstract (web summary)

A scalable communications protocol for transmitting spike-train data generated from multiple microchip sensors can be used with spiking neural network models for brain–machine interface and biosensing applications.

## Main

A wireless network of spatially distributed radiofrequency identification (RFID) sensors can collect real-time information by streaming data to a single receiver from many nodes simultaneously^1–4^. The received ensemble information can be used to make predictions about the trajectory of dynamically varying target environments in which the sensors are embedded. Such large-scale wireless sensor networks (WSNs)—composed of large populations of unobtrusive, battery-less, autonomous sensors—are of potential use in a wide range of applications, including environmental sensing and healthcare^5–9^. However, most commercial wireless RFID sensors lack a scalable network communication capability beyond a handful of devices.

In this Article, we report a large-scale WSN. Our approach is inspired by event-based, asynchronous, retina-mimicking dynamic vision sensor cameras, which convert time-varying illumination to sparse spike trains for energy-efficient, high-data transfer throughput at each pixel^10–12^. We apply the concept to a network composed of remotely powered silicon chips, which are assumed to be capable of converting recorded event data to time series of spikes. We then consider whether, in the case of event sparsity as with real biological neurons, it is possible to build a scalable wireless network of sensor neurons with improved energy efficiency and bandwidth usage.

Established communication approaches, such as versions of the random-access protocol^13–17^, show severe limitations in the transmission of spike-type event data from larger populations of wireless RFID-type sensors. This is due to the requirement of fixed timeslots and/or bandwidth allocation in the conversion of sparse binary events into packetized data, which undercuts the benefit of sparsity (when no meaningful events are detected, each node keeps transmitting and consuming radiofrequency (RF) bandwidth). Alternative approaches based on impulse radio have been developed, including the deployment of neuromorphic signal postprocessing. However, they also have limitations, such as the inability to identify specific sensors, leading to limited scalability and data loss from event collisions^18,19^.

A more efficient and scalable WSN can be created by transmitting binary spike events, after first digitally encoding the signal by a sequence of a predesigned spreading code. This code serves as the address for a given asynchronous sensor node, a particular instance of a more general address event representation (AER) protocol^20^, and allows an external receiver to recover and unpack spike-event data from each sensor in the network. Multiplying data on-chip with a unique pseudorandom number (PN), such as the Gold or Kasami code, has been theoretically analysed for an RFID tag^21,22^. A related idea involving an asynchronous AER approach specific to neural sensing applications was recently developed in the optical communication domain using a pulse interval modulation scheme. That work, which is based on computational simulations, suggests the possibility of transmission of up to 1,000-channel neuronal spike data via time-modulated light-emitting diode light^23^. A single-sensor wireless neuromorphic sensing system as an integrated circuit with compressive sensing and on-chip power-saving features has also recently been reported^24^. In that case, low-rate spiking data (from earthworms) encoding a peripheral neural waveform were transmitted using a 3-bit AER protocol for a single-point device.

We focus on the scalability of the WSN system and develop a hardware implementation of a communication chip in an application-specific integrated circuit (ASIC). In comparison with other wearable RFID sensors or related chip-scale RF-sensor concepts^25–33^—including our own recent work with RFID-type neural microsensors for brain implants^1,34,35^—this event-based RFID sensor network offers much larger scalability. Notably, the asynchronous sparse binary identification transmission (ASBIT) network protocol we develop faithfully preserves the timing information of the event detection and leads to an efficient means to transmit large amounts of event-driven data from a sensor population. Unlike many other communication schemes, the ASBIT method uses quasi-orthogonal codes—Gold code^21^—for spike-event communication to minimize communication errors and increase the capacity of a sensor network. Supported by experimental data with smaller populations of custom-designed silicon chips, we demonstrate in silico the scalability of the ASBIT protocol to thousands of sensor nodes at event error rates below 10^−3^. We also compare two different approaches to on-chip clocks in fabricated chips, necessary for on-board digital circuits, to optimize the network performance.

We then show how the population spike data received from such a wireless network lend itself to decoding by neuromorphic computing techniques to predict the dynamics of a target environment. We examine the effectiveness of spiking neural network (SNN)-based decoding methods in predicting state dynamics from spike-based ensemble recordings from the primate cortex. Related to efforts to build high-performance wireless brain–machine interfaces (BMIs)^36–39^, we use the ASBIT network to transmit over 8,000 channels of spiking neural data recorded from the primate cortex for decoding. We customize an SNN algorithm to accurately predict hand movement relative to the actual kinematics. While there are many other mathematical models used in computational neuroscience for decoding from populations of spiking neurons—ranging from the linear Kalman filter to convolutional neural networks—we believe co-design of an ASBIT-type wireless networking approach with neuromorphic computing can provide a low-latency, energy-efficient way to build large-scale WSNs.

## The ASBIT communication method

**Fig. 1:**
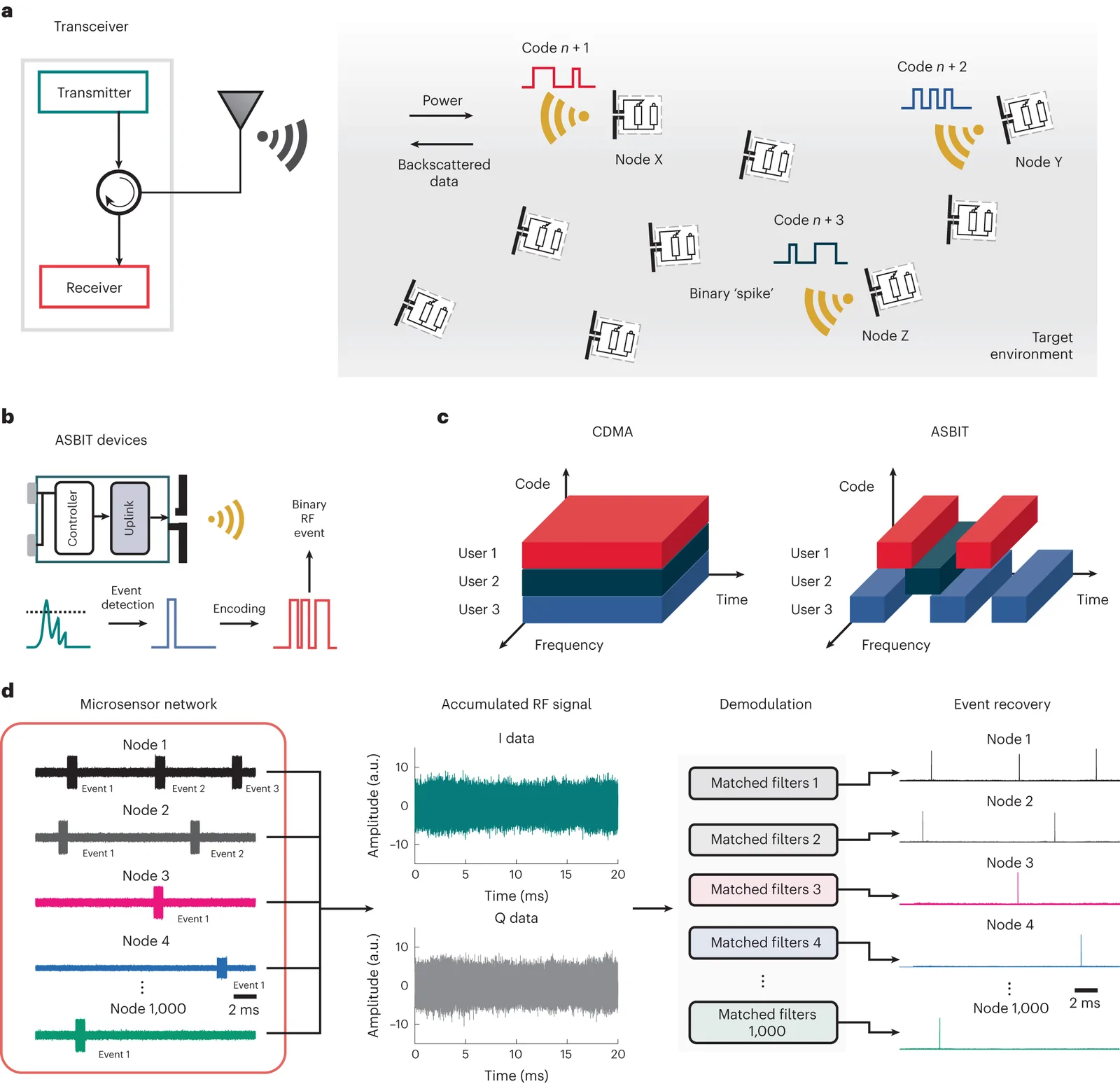
ASBIT-based communications system overview. **a**, The concept of a ‘detect and transmit’ microsensor communication network (asynchronous, sparse and binary). Each node transmits a detected sparse event via backscattering, encoded by a unique RF identifier sequence (for example, a Gold code), which is captured by a transceiver antenna. **b**, Schematic illustration of a generic event-detecting, neuronally inspired ASBIT sensor in which a binary signal is encoded with a pseudorandom code for RF transmission. **c**, Schematic highlighting the contrast in allocation of network resources (spectrum, code and timing) by the usual code-division multiple-access method and by the ASBIT method, respectively. The latter requires uplink bandwidth only when reporting a detected event. **d**, A flowchart illustrating the steps in unpacking the received ASBIT signals by demodulation (simulated data). Received IQ data (the graphs labelled ‘I data’ and ‘Q data’) show a raw superimposed signal from 1,000 nodes in this simulation, followed by the demodulation step where a battery of matched filters retrieves original events from each microsensor node.

Our ASBIT networking approach is illustrated in Fig. 1a, which shows wireless transmission by ensembles of wireless microchip sensors that acquire their power from, and deliver backscattered signals to, an external transceiver (here, near 900 MHz). We assume here that each silicon chip includes circuitry that converts any time-varying input signal to a spike train, not unlike the approach used in dynamic vision sensor cameras^10–12^ (Fig. 1b). In an ASBIT protocol, sparse asynchronous binary data in the form of spikes are further encoded with a unique on-chip RF identifier, such as the Gold code, before transmission to a receiver through backscattering. Unlike conventional code-division multiple access, the ASBIT scheme leverages statistical multiplexing of sparse data and information from event timing, analogous to a biological neuron firing^40,41^. As a consequence, efficient use can be made of key network resources in terms of the spectrum, code and timing (Fig. 1c).

By combining results of benchtop experiments on populations of microfabricated submillimetre CMOS chips with in silico simulations, we demonstrate how a single ASBIT link is scalable up to thousands of wireless sensors, where the degree of event sparsity sets an error-rate limit for the system. The serial steps to unpack aggregate signals in the process of RF demodulation at the receiver are shown in the flow diagram of Fig. 1d. The raw incoming RF data (the graphs labelled ‘I data’ and ‘Q data’) show the example of a simulated aggregate signal from 1,000 sensors where the superposition of many backscattering signals masks information from any individual sensor. However, if data on each chip are digitally encoded with a unique identifier, such as the quasi-orthogonal Gold code described and implemented below, any set of binary events across the entire sensor ensemble can be recovered by an appropriate demodulation technique, here using matched filters (Supplementary Note 1 and Supplementary Fig. 1). In the following, we first discuss the design of CMOS-based chips for testing the ASBIT protocol by explicitly considering two different methods for an on-chip clock to provide the timing for the on-board digital circuits. Namely, we compare the case of a free-running oscillator versus the case where the timing signals are derived by frequency downconversion of the ∼900 MHz baseband from an external transceiver unit. Further details of chip circuits and their characterization are given in Methods; relevant performance comparison between the two on-chip clock approaches is given in Supplementary Note 3 and Supplementary Fig. 5. The microdevices in this paper employ the near-field (inductively coupled) electromagnetic regime for signal and power. However, the ASBIT-based communication approach is quite general and applicable to the far-field or other communication modalities as well.

## On-chip free-running oscillators

**Fig. 2:**
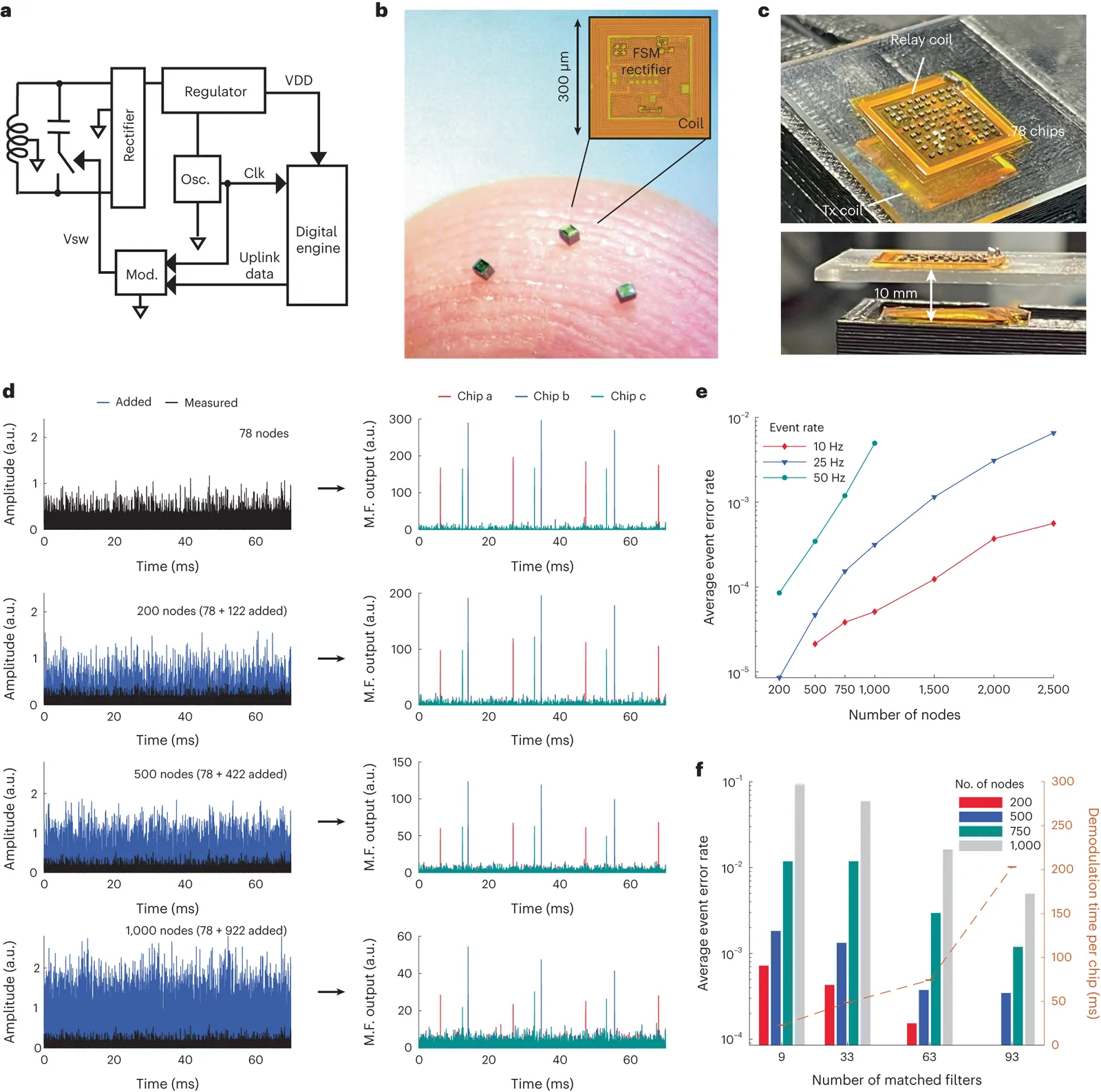
Prototype of ASBIT system-on-chip wireless ASIC with on-chip oscillator, coil antenna and digital logic circuit. **a**, Principal circuit blocks of the ASIC, with an on-chip antenna coil, rectifier, digital logic circuit and oscillator as the system-on-chip clock. **b**, Microphotograph of wireless ASIC prototype chips on a fingertip as an illustration of size, with inset showing the chip layout. **c**, Photograph of the benchtop three-coil antenna configuration in wireless experiments (through path 9 mm in air, 1 mm glass). **d**, Left, combining measurements with simulations to study the scalability of the ASBIT network, with initial backscattered signals measured from 78 fabricated chips (here, with an average SNR of 1.7 dB). A larger-scale network was generated by simulating data backscattered from the equivalent of 200, 500 and 1,000 sensor nodes. Right, transient outputs of the matched filters (labelled M.F.) for each case considered in **d** (78, 200, 500 and 1,000 nodes). The outputs from the matched filters for data received are shown for three randomly chosen chips, a, b and c, obtained using three different sets of matched filters that were individually calibrated for each chip. **e**, Average EER (*n* = 40, 6 s simulation epoch) as a function of the number of nodes added to the network. Each ‘background’ node detecting 10, 25 or 50 asynchronous events per second, respectively. Sparsity in event detection in the background enables a considerably larger number of nodes to operate in the network. **f**, Average EER as a function of the number of nodes and matched filters used for event recovery to account for the clock drift in each chip. The plot also shows how a longer demodulation time per node is required with an increased number of matched filters (dashed line; 1 s data). Osc., oscillator; clk, clock; vsw, switching voltage; mod., modulator.

The prototype wireless microchips with on-chip free-running clocks were designed to be submillimetre in size for potential use in body implants, such as in our previous work on neural implants^1,34,35^. Figure 2a shows an overview of the circuit blocks in the ultra-low-power, system-on-chip silicon die, including a low-voltage rectifier, a regulator, an oscillator, a generator for the Gold code address/identifier^21,42^, a digital finite-state machine and a binary phase shift key (BPSK) modulator for backscattering (see also Supplementary Fig. 1b,c for the chip layout). These communication chips were fabricated in TSMC’s 65 nm mixed-signal/RF low-power CMOS process with the chip footprint miniaturized to a grain-of-sand size of 300 × 300 µm (Fig. 2b). Constraints of small size and power, as well as maintaining circuit simplicity, make an on-board high-precision crystal oscillator or other advanced clock stabilization circuitry impractical. The simple free-running relaxation oscillator that we chose has an operational frequency near 30 MHz.

The inset microphoto in Fig. 2b shows that a considerable part of the chip area is occupied by the multi-turn power harvesting coil around the chip perimeter plus the associated rectifier and impedance matching circuits. By way of comparison, the digital finite-state machine (FSM), which houses the communication code, only requires an area of some 35 × 60 μm. For demonstration purposes, the communication circuit parameters were set to generate Gold-coded, backscattered transmission every 20 ms, corresponding to the case of an average event rate of 50 Hz. As a test of the receiver electronics, Supplementary Fig. 2 shows a clip of measured in-phase/quadrature (IQ) data from this wireless chip, the time window spanning the microsecond timescale of a Gold code packet, compared with matched filter waveform generated by a custom synthesizer that we developed for this purpose (for the details, see Supplementary Note 1, Supplementary Fig. 3 and Methods). As a practical matter, and to help make quantitative measurements of the wireless communication across a wide range of signal amplitudes for a population of chips, most of the benchtop tests were conducted using a three-coil near-field configuration in which a population of chips was encircled by an additional inductive relay coil in the same plane, separated by 10 mm from the transmitting (Tx) coil (photos in Fig. 2c)^1^. In RFID-type systems that use RF backscattering for communication, including our proposed ASBIT method, wireless transfer efficiency plays a central role in determining the signal-to-noise ratio (SNR). As the metric, we used the received signal strength indicator (RSSI), explained in Supplementary Note 2 and Supplementary Fig. 4.

We fabricated 78 wireless chips for the experiments, a practical number given limitations in a multipurpose wafer foundry run yet large enough for statistical performance analysis. We collected the backscattered signals from this population where the top left graph of Fig. 2d shows the received aggregate signal. Because of the on-chip Gold code identifier (and its correlation properties), very good autocorrelation across the full chip population was verified for the received packets with no measurable communication errors, as expected for such a relatively small network (Fig. 2d, top right row). Then, to extrapolate the performance of the ASBIT protocol for much larger chip populations (nodes), we synthetically added ‘background signals’, which are the previously collected data from actual chips, results being shown in the lower three graphs of Fig. 2d. To do this, we divided the chips into two groups, namely, 40 ‘target’ chips and 38 ‘background’ chips, and randomly added signals from the latter chips to artificially multiply the total population. For instance, when simulating 1,000 nodes, we selected each of the 38 chips 922 times, with the replacement, in a random sequence and added the packets from a given selected background chip to the original data (already containing backscattering data from the 78 chips). This method allowed us to analyse the communication fidelity within the target group while ensuring that no additional events were generated by the ‘target’ chips themselves in assessing the contribution of a large number of nodes in the background. The examples in Fig. 2d of amplitude recovery from matched filter outputs demonstrate how the ASBIT method can differentiate a specific target packet even when multiple packets undergo interfering collisions. Even when 1,000 nodes were included in the network, whereby the original signal from a given chip is entirely obscured by the background, we were able to reliably detect the specific event in the output of the matched filter.

In the ASBIT concept, a bit ‘one’ represents an event detected at a given sensor node, accompanied by the particular Gold code. To quantify the accuracy of data transmission, we solved for the event error rate (EER), where a missing event or an instance of false detection was considered an error. Without loss of generality, we assumed a nominal duration of each event (the bin size) of 1 ms, a choice appropriate for the neural application example in the last part of this paper. Figure 2e shows the average EER of 40 target chips at an average SNR of 1.7 dB as a function of the number of other chips contributing to the background interference. We calculated that at the average event rate of 50 Hz, the ASBIT protocol can accommodate 750 nodes in achieving an EER of 1.19 × 10^−3^, a value acceptable for many applications, including for BMI use. We also tested the impact of different event rates for the background nodes to evaluate how sparsity affects the communication quality of the ASBIT protocol. Figure 2e suggests that, without degradation of the EER, we can accommodate a substantially larger number of chips, up to 2,500, with sufficiently sparse events while demonstrating the protocol in utilizing sparsity without any need for additional adjustments or further programming. The ability of each node to communicate with a single external receiver (or equivalently a single RFID reader) without the need for complex on-chip programming helps to keep the system architecture simple.

A penalty for using a miniaturized free-running oscillator in our microchip is it being subject to a clock drift of approximately ±1,000 ppm over time, the drift caused by fluctuations in chip voltage supply, ambient temperature and other factors. The drift affects the waveform of the Gold code packet so that using a matched filter designed for a specific frequency can lead to inaccurate correlation values. To address this issue and to maintain the low EER values, we applied multiple sets of matched filters (Fig. 2f). The cost of using multiple matched filters is the increase in the demodulation burden, as shown in Fig. 2f, which may be problematic in applications requiring low latency. (For the description of the computational pipeline for sensor ensemble RF demodulation, please refer to the ‘Demodulation of ASBIT signals’ in Methods.)

## Using an RF carrier downconversion approach

**Fig. 3:**
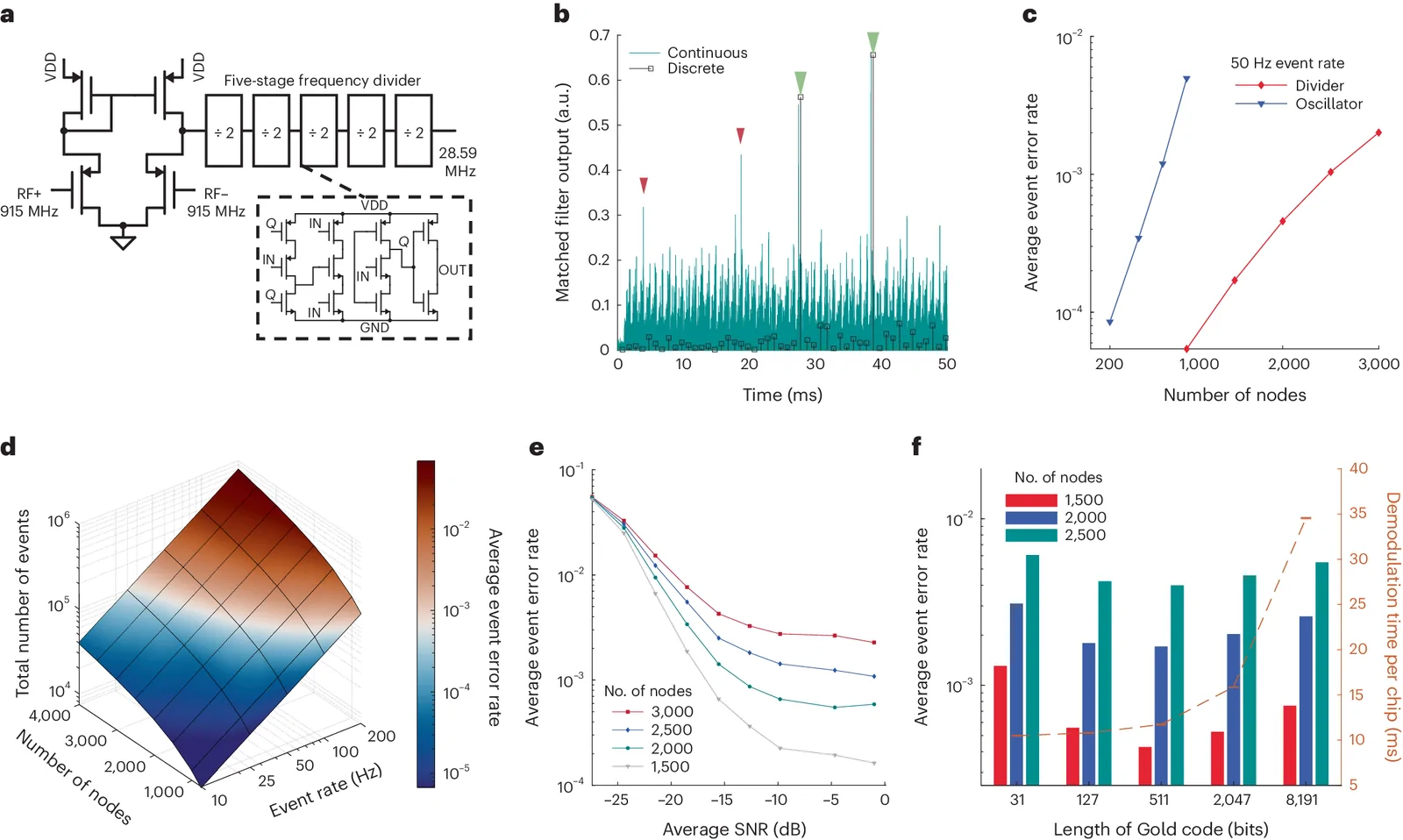
An improvement of EER and network capacity for microchips with clock frequency divider. **a**, Circuit diagram of a differential amplifier with multiple-stage true single-phase clock dividers to derive the clock frequency from a downlink RF energy source in place of an on-chip oscillator. **b**, Comparison of matched filter outputs acquired from continuously received data in case of an on-chip oscillator versus corresponding discrete RF data for chips incorporating a frequency divider for the clock (selected by estimating timeslots of a target wireless chip). The red triangles show instances that can cause false detection, in this case, of a continuous matched filter output, while the green triangles indicate a correct event detection. **c**, Improved EER for the ASBIT chips with a clock frequency divider; here, the receiver is detecting 50 events per second over 6 s, as a function of the number of nodes in the network (average SNR of 1.7 dB). **d**, Heatmap representation of the overall network capacity, quantified as total number of transmitted events per second and the averaged EER for 22 selected nodes (right-hand colour bar) as a function of the number of nodes and the event rate (SNR = 1.7 dB, 30 s simulation time). **e**, Variation in average EER in relation to both network size and SNR, the latter varied by adding Gaussian noise; 6 s simulation time. **f**, Dependence of average EER on the number of nodes and the length of the Gold code at SNR of 3.23 dB, for identifying an optimal code length for network performance. The plot also shows demodulation time per chip for a 1 s piece of data, showing the increase when increasing the length of the Gold code (average event rate is 50 Hz; transmission window is 6 s). VDD, voltage supply; GND, ground; Q, inverted output node.

We next investigated the use of the RF downlink delivering power as a suitable frequency reference in circumstances where this might be advantageous for our wireless sensor network concept. Using the baseband RF for timing has been demonstrated, for example, for RFID tags where the incoming RF frequency (in our case, ~900 MHz) is divided by a fixed integer to generate a lower frequency clock^43–45^. One particular choice is a multiple-stage true single-phase clock frequency divider^46^, shown schematically in Fig. 3a. The approach can offer the benefit of negligible clock variance and near-independence from energy harvesting efficiency, however at the expense of increased power consumption as depicted in Supplementary Note 3 and Supplementary Fig. 5. Note that a frequency divider approach does not imply network synchrony since the phases of clocks of individual chips will differ due to phase lags arising, for example, from the random start-up of each chip’s starting circuit.

We fabricated a set of chips in which the frequency divider circuit replaced the free-running oscillator as part of the same prototype ASBIT communication test chip. In benchtop measurements, we could obtain the timing sequences across the entire chip population as the demodulation step for the backscattered signals was now simpler in the absence of frequency shifts. In Fig. 3b, we illustrate how the application of matched filters within a predefined time window enables the direct exclusion of false detections, leading to an improved EER. One additional consequence is that the ASBIT protocol can be expected to accommodate even larger numbers of network nodes than in the case of an on-chip oscillator. We examined this network scalability by again using the method to synthetically add real-chip background signals to the network, as in the case of the free-running chip population above. Here, we used a slightly smaller population of 65 chips, the number limited due to postprocessing loss during dicing and handling. The results in Fig. 3c show how this RF-clocked ASBIT network can accommodate an ensemble of up to 2,500 microsensors while maintaining an EER of 1.03 × 10^−3^ for an SNR of 1.7 dB (again assuming an average event detection rate of 50 Hz for each node). This represents more than a three-fold increase, as compared to the free-running on-chip oscillator case.

As a multidimensional summary of the EER analysis using the fabricated chip population, Fig. 3d shows how the network capacity is influenced by the average event rate across all nodes, here over a range of sensor nodes from 500 to 4,000. The heatmap was generated by multiplying the statistical event (‘firing’) rate by the number of nodes in evaluating the EER for the full network. As expected, the EER increases both with increasing event rate and the number of nodes. Overall, the aggregate event sum is what mainly determines the network communication performance and capacity. Here, approximately 100,000 events were collected per second, achieving an EER below 10^−3^. This result suggests that the ASBIT protocol can be adjusted to be quite flexible with a very simple scaling rule: a smaller number of sensor nodes allows for high event activity rates, while a greater number constrains the network to sparser event rates.

Emulating real environmental effects, we further evaluated the effect of SNR on the ASBIT network performance by adding Gaussian white noise numerically to the experimentally acquired signals with the result shown in Fig. 3e. To account for the additional noise, we recalculated the average SNR by computing the ratio between the RSSI of each packet and the background noise level. As seen in Fig. 3e, the EER increases at low SNR values while the achievable error rate in a smaller network is considerably lower for the same SNR. For instance, for 1,500 nodes an SNR of only −16.77 dB is needed to achieve an EER below 10^−3^. Thus, the ASBIT method can achieve acceptably low values of EER even when the backscattering amplitude is lower than our experimentally measured RSSI of −74.45 dBm, as long as the number of nodes is limited to approximately the one-thousand range. The robustness to noise stems mainly from the effective bit protection provided by our Gold code strategy. For more information on coding gain, please refer to Supplementary Note 4.

Finally, we analysed the ASBIT network by varying the length of the Gold code in simulations. Unsurprisingly, shorter codes offer lower coding gain and poorer auto- and cross-correlation properties. At the other end, very long codes can result in severe interference between backscattered signals, which degrades the EER. Figure 3f summarizes this relationship and shows that the optimal Gold code should be approximately 511 bits, the actual value we chose in our circuit co-design for the microfabricated chips. Longer code sequences also require more complex matched filtering, thereby leading to an increase in demodulation time (see the dashed line curve in Fig. 3f). Through simulations, we found that, for the case of a 511-bit Gold code, the demodulation time per a target node for a 1-second slice of data was only 12 ms, sufficient for many applications. As such, the drift-free frequency divider approach shortens the demodulation time as only three matched filters are needed to account for sampling the phases (see the comparison of the steps in the demodulation event recovery process for the on-chip oscillator and the frequency divider cases in Supplementary Fig. 6). In Fig. 3f, we synthesized waveforms for all packets due to the fixed length of the Gold code in the experimental chips, assuming an average SNR of 3.23 dB.

For practical applications, it is important to consider the design of wireless power transfer (WPT) for any large-scale battery-free sensor network. As a case example relevant to brain implants, where RF energy in the near-field (inductively coupled) regime must travel through a considerable thickness of lossy tissue (the scalp, skull and dura), we show, in Supplementary Note 6 and Supplementary Fig. 9, an approach using additional thin planar relay coils for an enhanced WPT system. On the other hand, given the scalability of the ASBIT method, the performance of a wireless network is not limited by data bandwidth in the first instance, especially for sparse events, such as for typical neuronal firing rates. Rather, the number of sensors is likely limited by the WPT efficiency and the electromagnetic design of the energy delivery. When limited by regulatory considerations—for example, specific absorption ratio with increasing Tx power—it will be necessary to further reduce the sensor chip power consumption, such as by transitioning to more advanced CMOS process nodes or utilizing an additional power source to deliver extra energy^23^.

## Transmission and decoding by spiking neural network

As an application example of the scalability of the ASBIT method, we considered the dual challenge for a mobile BMI to perform increasingly complex tasks: wireless transmission of data recorded from thousands of points in the brain and the subsequent predictive neural decoding to operate an assistive device. The question can be posed more generally, namely, as the co-design of means for data transmission from any sets of spatially distributed autonomous sensors embedded in a dynamical environment to the choice of the computing paradigm to predict its anticipated trajectory. We reasoned that, as an event-based method transmitting data in the form of spikes, the ASBIT technique might be well matched with large-scale decoding tasks by the use of neuromorphic computing techniques. Neuromorphic computing, itself inspired by the brain, has recently developed to the point where low-power, portable hardware capable of executing sophisticated models of SNN at very low latency has become available^47^. At the same time, many mature methods exist in computational neuroscience for decoding neural data for BMI purposes, ranging from classical linear techniques (Kalman filter) to deep machine learning, including our own recent work^48,49^.

**Fig. 4:**
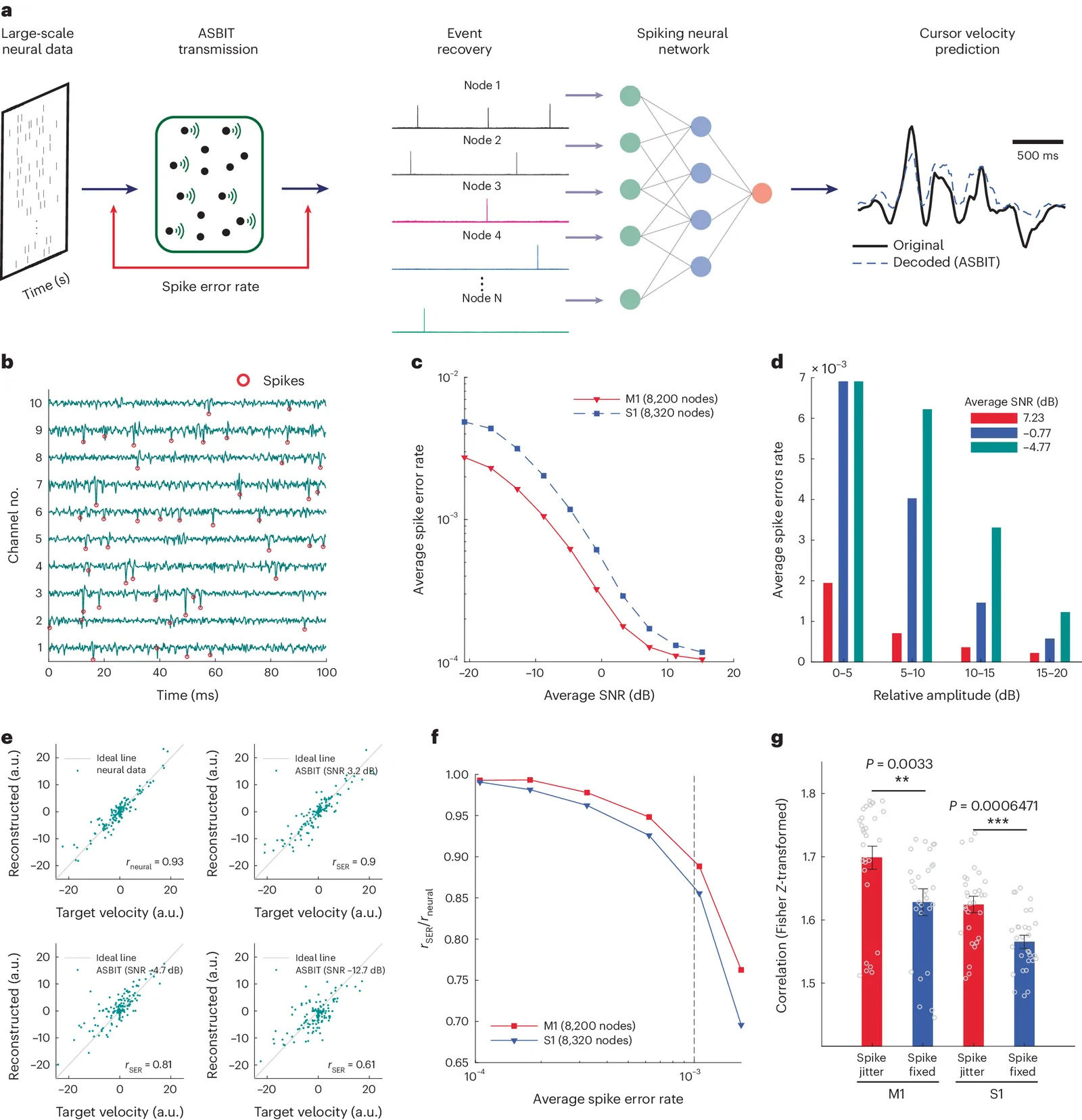
Transmitting and decoding over 8,000 channels of spiking neural data. **a**, Schematic illustration of the wireless transmission of neuronal spike events using the ASBIT protocol. Received signal at an external wireless hub is demodulated to extract spike data from each node as input to an SNN-based decoder to predict cursor velocity. **b**, Example of raw broadband data from auditory cortex^48^, sampled at 30 KSa s^−1^ from ten channels. The red circles show neural spikes detected by thresholding. **c**, Spike error rate versus SNR, the latter controlled by adding Gaussian white noise to M1 data (8,200 channels, 164 neurons × 60 datasets) and S1 data (8,320 channels, 52 neurons × 160 datasets). Large-scale neural data were synthesized as described in Supplementary Fig. 7; the data were obtained from refs. ^50,51^. **d**, Average spike error rates computed based on the backscattering amplitude indicate that the average SNR and amplitude are the two dominant factors affecting SER. **e**, The relationship between target *x* velocity and reconstructed *x* velocity from the SNN neural decoder. The top left graph shows the reconstructed velocity using neural data collected from a wired system, while the other graphs show the decoded velocity using spike data transmitted through the ASBIT protocol at different SNRs (200 data points). The grey line represents the ideal line where the target and reconstructed velocity exactly match. **f**, Dependence of neural decoder performance on average SER, showing correlation (*r*_SER_) between cursor velocity and velocity reconstructed by using spike data transmitted through the ASBIT. The value for *r*_SER_ is normalized to correlation (*r*_neural_) obtained by using original spike data. Each correlation value is averaged across five folds. **g**, Effect of spike timing jitter on the neural decoder’s performance, highlighting the advantage of transmitting neural spikes to preserve precise spike timing information (here, SNR = 3.23 dB). For statistical analysis, correlation values are obtained in ten-fold cross-validation^60^ and Fisher *Z*-transformed to approximate the normal distribution. Asterisks indicate the *P* values obtained from two-sided paired *t*-tests. Error bars present mean values, and whiskers show ±standard error (*n* = 30, repeated ten-fold cross-validation, three trials).

Here, we assessed the scalability of the ASBIT network and determined the acceptable spike-event rate (SER) that can achieve the desired SNN neural decoding performance for hand velocity prediction in controlling a cursor. We used open source non-human primate data recorded from the primary motor cortex (M1) and its mapping on the somatosensory cortex (S1)^50,51^, both associated with hand movement intentions, to synthetically scale the spiking data to thousands of spiking neurons. In this context, Fig. 4a illustrates schematically how a population of hypothetical wireless microsensors detects neuronal spikes and transmits the spike ensemble events through the ASBIT protocol. The schematic follows the path of the signal flow from the sensors implanted in or on the cortex to the external SNN neural decoder. After demodulation of the Gold-coded RF data from a population of sensors, the decoder output is translated into hand-kinematic commands. The SNN decoder, described below and in Methods, accepted the binary spike data as the input and generated a reconstructed cursor velocity^52–55^. Since the neural signals, the ASBIT protocol and the SNN model are all spike-based, a seamless integration of these elements is both natural and efficient.

The firing of neuronal action potential spikes is inherently sparse^56,57^, shown in the example of Fig. 4b of raw data recorded in our laboratory from the auditory cortex of a non-human primate by intracortical microelectrode arrays^48^. Similarly, in ref. ^50^, the mean firing rate of 164 neurons in the primary motor cortex data during the centre-out hand-reaching task is shown as 9.2 Hz. Given this sparsity, we simulated a large-scale wireless BMI by including the wireless ASBIT protocol in the loop where the detected spike events are transmitted as Gold-encoded packets for each neuron. Since large-scale experimental neural data are currently unavailable, we used the limited open source data to synthesize equivalent spiking data from 8,200 separate nodes. Specifically, we transmitted 50 trial datasets from 164 electrodes simultaneously, as detailed in Supplementary Fig. 7. To generate the set of 50 × 164 = 8,200 required Gold code packets, we developed a custom algorithm for synthesizing a matched filter that allowed for accurate modelling of the packet from chips as well (see Supplementary Note 1 and Supplementary Fig. 3).

We first analysed the SER in ASBIT transmission, where—analogous to the EER—a missing target spike or a false detection counts as an error within a time bin of 1 ms. For this, we prepared an 8,200-channel M1 neural dataset for a 24-s and an 8,320-channel S1 dataset for a 19-s data acquisition window. We calculated the average SER of the received spike trains across the 8,000-plus sets of nodes as a function of SNR with the result in Fig. 4c. Assuming an average SNR = −0.77 dB, not unrepresentative of animal experimental situations, we computed an average SER of 3.23 × 10^−4^ in M1 data and 6.12 × 10^−4^ in S1 data for the entire network. Meanwhile, to emulate the near–far electromagnetics problem in an implant, where a given microchip closer to the Tx coil generates a stronger signal, we varied the backscattering amplitude by up to a 20 dB difference in SNR across sensor microchips. Figure 4d and Supplementary Fig. 8 show the dependence of the average or individual node’s SER, respectively, on the relative SNR, indicating that the backscattered amplitude of a specific node dominantly determines the SER of that node. This should be similar in far-field or other regimes where the external power source projects a spatially inhomogeneous electric or magnetic field.

For the neuromorphic deep-learning approach, we trained an SNN model to reconstruct the cursor velocities using the movement-intention-evoked intracortical spike data. We compared the outcomes for the hypothetical case of ideal, lossless wired electrodes (data also synthesized from the open source data) with the case where such data were transmitted through the wireless ASBIT chain. Figure 4e depicts the relationship between the actual kinematic *x*-component of the hand velocity towards a target and the reconstructed velocity using cortical spike data. The top left plot presents the prediction using neural data without any spike loss, showing a linear relationship between the kinematic and the reconstructed velocities. The other plots compare the kinematic velocity with the prediction obtained from spike data transmitted through the ASBIT protocol for different SNRs affecting the number of spike errors. In the low SNR regime, the data points deviate from the ideal line and the decoding performance degrades.

To further quantify the decoding performance, we computed the correlation coefficient between the original velocity and the reconstructed velocity on testing sets^50,58^, as described in Methods. We obtained the correlation coefficient using the wired neural spike signals, referred to as *r*_neural_, and using the wirelessly transmitted neural signals, denoted as *r*_SER_. Since the ASBIT link may introduce spike errors, we expect *r*_SER_ to be lower than *r*_neural_. In the absence of spike errors, average results for *r*_neural_ were 0.9324 for M1 data and 0.9297 for S1 data, both of which were obtained using a five-fold approach. All correlation values obtained in the five-fold cross-validation are presented in Supplementary Table 2. Figure 4f shows the ratio of *r*_neural_/*r*_SER_ as a function of the SER. The results suggest that, even with an SER of 10^−4^, the decoding performance of the ASBIT system (*r*_SER_) is comparable to that achieved by wired microelectrode arrays (*r*_neural_), as *r*_SER_/*r*_neural_ approaches unity. Even for an SER of 10^−3^, the wireless ASBIT link can still achieve 88.8% and 85.4% of the decoding performance compared to an equivalent wired system for M1 and S1 data, respectively. Note that we assumed the simultaneously transmitted 50 datasets for M1 as well as the 160 datasets for S1 to be independent of each other. Yet, since many neurons in the primate brain encode shared information and were not independent of each other, we anticipate that a large-scale ASBIT-based wireless neural interface, when implemented as a chronic implant, is likely to have better decoding performance, *r*_neural_, in being more robust to spike errors.

We also conducted an additional neural decoding experiment to investigate the importance of preserving high-resolution spike timing information through the ASBIT protocol. We first introduced random jitter to the spike timing (up to ±25 ms) in each SNN training epoch of recorded neural data transmitted via the wireless link. The random jitter was introduced to mimic the stochastic character of neurons and to enable the SNN model to be trained across many different temporal patterns of spike trains. We then compared the correlation values with and without jittering, as shown in Fig. 4g. We performed Fisher *Z-*transformation on the correlation for statistical analysis and found that the average correlation was in fact higher with spike jittering for both M1 and S1 data. A paired *t*-test showed a statistically significant difference between the two cases in both datasets (*P* = 0.0033 in M1, *P* = 0.00007 in S1). These results suggest that preserving spike timing information at high resolution is important in building an SNN-based neural decoder that is robust to spike errors and to inherent stochastic activity in neurons, leading to improved decoding performance.

## Conclusions

We have reported a communication approach for large-scale wireless asynchronous microsensor networks that transmit binary events from thousands of local nodes with spectral efficiency and at low error rates. The received data serve as input for the reconstruction of the dynamical states of the environment in which the sensors are inserted. Our ASBIT approach, with each node reporting binary events above the threshold, is inspired by principles governing information processing in the brain, including intrinsic sparsity of single neuron activity. We have demonstrated the ASBIT protocol on submillimetre-sized silicon chips with low-error-rate transmission and robustness from collisional interference, using an event recovery method based on matched filter techniques. We investigated two different on-chip timing strategies: a free-running oscillator and a clock frequency divider. The former allows low-power autonomous operation of microsensors, and the latter enables further system scalability in reducing transmission error rates and latency at the expense of higher power consumption. (A description of how our method compares with other relevant communication techniques is given in Supplementary Note 5 and Supplementary Table 1.)

We performed simulations on the scalability of an ASBIT network in the joint BMI context of spike-based wireless transmission and SNN model neural decoding. We used sets of experimental neural data recorded from the primate cortex, synthetically scaling up to 8,320 independent spiking channels, in decoding cortical state dynamics for the reconstruction of cursor movement intention. Although our work mainly focuses on the ASBIT communication method, it is important also to anticipate practical challenges associated with wireless RF power transfer in building a large-scale wireless neural or other body-implanted sensor network. For medical use, further advances in ultra-low-power sensor design with power consumption in microwatt range can further benefit from advanced semiconductor process nodes. To ensure safe scaling to thousands of nodes, a more refined resonance tuning mechanism is beneficial to reduce energy waste throughout the network. Additional electromagnetic design options might include deployment of a distributed RF powering system when access to a wider area of the brain cortex is sought while ensuring compliance within specific regulatory absorption ratio limits.

Our approach should also apply to the design of other large-scale wireless sensor networks where, using a spike-based binary approach, the transmission at low error rates combined with neuromorphic data analysis can be useful in predicting the dynamics of a complex heterogeneous target environment.

## Methods

### The ASIC chip design and functional validation

The prototype microchips, designed to operate in the ASBIT transmission mode, were designed by partly leveraging our previous work using the TSMC 65 nm mixed-signal RF low-power CMOS foundry process. A diode clamp provided over-voltage protection from the energy harvesting process for the circuits, while a low-dropout regulator helped to stabilize the voltage supply for the on-chip oscillator. The Gold code generator and digital processor were implemented on an FSM, running at nominally 10 MHz, one-third the rate of either the free-running oscillator or the frequency divider output. We tested the silicon chips received from the foundry under fully wireless conditions in the laboratory using a software-defined radio (the SDR Model ‘Raptor’ by Rincon Research or FMCOMMS3 by Analog Devices) and a single-turn polyimide printed circuit board, which included the transmitter Tx coil and relay coil (both 9 × 9 mm). The SDR, together with Analog Devices AD9361 transceiver chips and Zynq SoCs, generated an RF baseband carrier at 915 MHz, which was further amplified by an RF power amplifier (ADL5605-EVALZ, Analog Devices). An RF surface acoustic wave duplexer (D5DA942M5K2S2, Taiyo Yuden) isolated the backscattered signals from the downlink carrier. The SDR performed amplification of backscattered signals, downconversion from 945 MHz to DC and finally the analogue-to-digital conversion at 30 MSa s^−^^1^ (12-bit), as in Supplementary Fig. 2. The digitized IQ data were then ported to a personal computer for offline ASBIT demodulation (computation in MATLAB/Simulink).

### Modelling of backscattering signals from RFID microsensors

Equations (1)–(6) and Supplementary Fig. 3 illustrate a stepwise simulation process of RF signals from a microsensor system utilizing backscattering modulation, where the RF transceiver hub emits a continuous carrier wave as

1 $${x}_{\rm{c}}\!\left(t\right)=\mathrm{Re}\left[{A}_{\rm{Tx}}{\rm{e}}^{\,j\,2\uppi \,{f}_{\rm{Tx}}t}\right]$$

where ${A}_{\rm{Tx}}$ and ${f}_{\rm{Tx}}$ denote the amplitude and the frequency of the Tx carrier, respectively, while *t* indicates time. The incident backscattering waves can be expressed as

2 $$\begin{array}{c} {s_{\rm{bck}}(t)=\mathop{\sum}\limits_{n=1}^{N_{\rm{s}}} S_n p_1\left(t-n T_{\rm{s}}\right)} \\ \\ \,\,{\mathrm{where} \,\, p_1(x)= \begin{cases}1 & \mathrm{if}-\!T_{\rm{s}} / 2 \leq x \leq T_{\rm{s}} / 2 \\ 0 & \mathrm{otherwise}\end{cases}}\\\end{array}$$

${S}_{n}$ is the coded symbol sequence that takes either the value +1 or −1, ${N}_{\rm{s}}$ is the number of symbols in the sequence and ${T}_{\rm{s}}$ is the duration of a symbol. In the proposed ASBIT protocol, the symbols are encoded in BPSK modulation using a system clock (${f}_{\rm{clk}}$) that is three times faster than the symbol rate^1^; thus ${f}_{\rm{clk}}=3/{T}_{\rm{s}}$. Therefore, ${s}_{\rm{{BPSK}-{bck}}}$ is defined as

3 $$\begin{array}{r} \\s_{\mathrm{BPSK}-\mathrm{bck}}(t)=\mathop{\sum}\limits_{n=1}^{N_{\mathrm{s}}}{\mathop{\sum}\limits_{m=1}^{3N_{\mathrm{s}}}{S_n}}p_1\left( t-nT_{\mathrm{s}} \right) \times S_{\mathrm{clk}}p_2\left( t-mT_{\mathrm{s}}/3 \right)\\ \\ \,\,\mathrm{where} \,\,\, p_2(x)=\begin{cases}1& \,\,\mathrm{if} -\!T_{\mathrm{s}}/6\le x\le T_{\mathrm{s}}/6\\0& \,\,\mathrm{otherwise}\\\end{cases}\\\end{array}$$

${S}_{\rm{clk}}$ is the clock sequence, which also takes either the value +1 or −1. In our case, a chip generates backscattered data by modulating its antenna impedance between two states and the modulated backscattering signal can be approximated as

4 $${x}_{\rm{bck}}=\mathrm{Re}\left[{A}_{\rm{bck}}\times {s}_{\rm{{BPSK}-{bck}}}(t){\,\times \, {\rm{e}}}^{\;j(2\uppi {f}_{\rm{Tx}}t+\phi )}\right]$$

Here, ${A}_{\rm{bck}}$ is the backscattered amplitude determined by modulation depth and backscattering cross-sections, and $\phi =\frac{2\uppi }{\lambda }D$ is the phase delay^21^. *λ* and *D* denote the wavelength and the distance between the transceiver hub and the ASBIT chip, respectively. A duplexer at the transceiver isolates ${x}_{\rm{bck}}$ from the downlink carrier from the SDR capturing the backscattering signal. After a low-noise amplifier, a downconversion with a conversion factor ${\alpha }^{{\prime} }$ is performed at ${f}_{\rm{Tx}}$ + ${f}_{\rm{n.{clk}}}$ (nominal clock frequency, 30 MHz) to yield

5 $$\begin{array}{l}y\!\left(t\right)={\alpha }^{{\prime} }G{A}_{\rm{bck}}{s}_{\rm{{BPSK}-{bck}}}\left(t\right)\times\left[ \cos \! \left({2\uppi\, f}_{\rm{n.{clk}}}t-\phi \right)\right.\\\left.-j\sin \! \left({2\uppi\, f}_{\rm{n.{clk}}}t-\phi \right)\right]+\omega (t)\end{array}$$

where *G* is the low-noise amplifier gain in the SDR and the noise signal *ω*(*t*) is modelled as a zero-mean complex additive white Gaussian noise process. The received signal is then passed through an analogue-to-digital converter (ADC), whose output sequence $y\!\left(v\right)$ can be defined as

6 $$\begin{array}{l}y\left(v\right)={A}^{\prime}{s}_{{\rm{BPSK}}{-}{\rm{bck}}}\left(v\right)\times\left[\cos\left({2\,\uppi\,{f}}_{{\rm{n}}.{\rm{clk}}}v+{\phi}^{\prime}\right)\right.\\\left.\qquad\quad-j\sin\left({2\uppi\, f}_{{\rm{n}}.{\rm{clk}}}v+{\phi}^{\prime} \right)\right]+\omega (v)\end{array}$$

where $v=1,\,\ldots \, ,{N}_{\rm{sc}}{N}_{\rm{S}}$, which is the received signal sampled at time instants $t=\nu {T}_{\rm{s}}/{N}_{\rm{sc}}$. Here, ${N}_{\rm{sc}}$ is the number of ADC samples per coded symbol and ${A}^{{\prime} }={\alpha }^{{\prime} }G{A}_{\rm{bck}}$. Due to asynchronous properties in our network, the ADC sampling also can affect the phase of the received signal; thus ${\phi }^{{\prime} }=\phi +{\phi }_{\rm{ADC}}$ where ${\phi }_{\rm{ADC}}$ is the sampling phase. According to equation (6), the received signal is equivalent to the downconverted ${s}_{\rm{{BPSK}-{bck}}}(v)$ with an amplitude of ${A}^{{\prime} }$ and the phase shift of ${\phi }^{{\prime} }$. We performed downconversion on ${s}_{\rm{{BPSK}-{bck}}}(v)$ to model the received signal in our simulation while also including various amplitudes ${A}^{{\prime} }$, clock frequencies ${f}_{\rm{clk}}$ and phase shifts ${\phi }^{{\prime} }$, which collaboratively determine the waveform of received signals. Also, this downconverted ${s}_{\rm{{BPSK}-{bck}}}(v)$ is directly employed as a matched filter during the demodulation step. When simulating multiple nodes (*N* microsensors in the network), the received signal $Y\!\left(t\right)$ from the entire network was modelled as a superposition ${y}_{i}(t-{\tau }_{i})$ where *i* = 1, …, *N* and ${\tau }_{i}$ denotes the initial delay time of backscattering.

### Encoding using the Gold code

In the ASBIT protocol, each sensor chip has its unique Gold code identifier, that is, one from the many random sequences, often called PN sequences. Contrary to this designation, the Gold code is, however, predictable and reproducible. Among such PN sequences, maximum length codes are the largest codes that can be generated by a shift register and have a period of 2^*m* ^− 1, where *m* is the length of the shift register. Gold codes are generated from XOR multiplication of a preferred pair of maximum length codes and, in given *m*, the set of Gold codes is made up of 2^*m*^ + 1 codes in a total of length 2^*m* ^− 1. Gold codes are suitable for spread spectrum systems and are often characterized by their auto- and cross-correlations. The selection of the length of the Gold code is important because the length sets bounds on network capacity and its auto- and cross-correlation properties. The microsensors in the ASBIT network generate an ${S}_{n}$ using the Gold code generator in the on-chip digital FSM, which is encoded into BPSK, hence yielding ${s}_{\rm{{BPSK}-{bck}}}(t)$. This is the ${s}_{\rm{{BPSK}-{bck}}}(t)$, which is backscattered whenever the sensor front end detects an event.

The physically unclonable function (PUF) was generated by intrinsic 65 nm CMOS fabrication process variations and unique to each chip (see also Supplementary Fig. 1). We implemented a circuit design that utilized a 13-bit PUF as the seed for our transmitted 511-bit Gold code sequence. By allowing each chip to synthesize up to 8,191 bits of Gold code, we were able to increase scalability and reduce the chance of PUF collision. However, to reduce mutual interference and achieve better performance in larger networks, we used only 511 bits out of the 8,191 bits of Gold code sequences. This decision was based on our analysis of the optimal length of the Gold code as described in the text.

### Demodulation of ASBIT signals

To demodulate the received ASBIT coded data stream, summarized in Supplementary Fig. 6, we used multiple sets of matched filters for the received signal $Y\!\left(t\right)$. A set of matched filters for the target node was designed by using the prediscovered digital Gold code accounting for the phase shift ${\phi }^{{\prime} }$, the clock frequency and the clock drift over time. Using those parameters, we synthesized multiple functions ${y}_{i}(t)$ following equation (5) and directly used these waveforms as matched filters. Note that we needed to separately build matched filters for I data and Q data and then to multiply these two at each time point to build a whole robust mechanism for phase cancellation. The first step of the demodulation was the clock recovery process where we synthesized matched filters assuming various values of ${f}_{{clk}}$, ranging from 27 to 33 MHz, and applied them to short clips of incoming data $Y\!\left(t\right)$ to check the matched filter output dependence on ${f}_{{{\mathrm{clk}}}}$. By checking the maximum value of a matched filter output, we could find whether the target chip generated backscattering pulses (or not) and could thus solve its clock frequency. Using the discovered clock frequency, we synthesized a smaller set of matched filters, although clock drift still needed to be accounted for. For example, for clock drift of 1,005 ppm (±30 kHz), we used 93 matched filters (3 phase variants × 31 clock points). In the case of the frequency divider scenario, on the other hand, one only needed up to three matched filters for correcting phase variants as the clock frequency was now well-defined (zero drift assumed). To accelerate the demodulation process, we can first find the exact time slot of a given node and solve ${\tau }_{i}$ and then apply matched filters to signal function $Y\left(t\right)$ by a discrete timing approach (which can be done in a phase recovery process also by using a short piece of $Y\!\left(t\right)$). After synthesizing matched filters, we applied these to $Y\!\left(t\right)$ to get multiple matched filter outputs, which were summed to get their combined continuous or discrete matched filter outputs. Finally, events reported by any sensor node could be detected by thresholding the combined matched filter output. We determined the threshold based on the root mean square of the matched filter output (which makes the process adaptive to the properties of the network).

### Decoding of neuronal data using spiking neural networks

We evaluated the performance of population neural decoding using an SNN algorithm from the Lava-dl library^52,59^ and assessed how the transmission fidelity of neural spiking signals in the ASBIT protocol affected the neural decoding. We chose an SNN technique as opposed to another machine learning approach based on intuition outlined in the text, but also as it can be readily implemented on recently developed portable Loihi2 neuromorphic hardware for real-time processing^47^ suitable in a BMI application.

Our SNN model comprises three dense neural network layers and a sigma–delta neuron input and output layer. The model used 650 ms of spike data at 1 ms resolution to estimate the velocity of the cursor in the *x* direction. To account for the inherent stochastic nature of neural activity, we randomly shifted the spike times (up to ±25 ms) during each training epoch, a process known as spike jittering. After spike jittering, we binned the spike data into non-overlapping 25 ms intervals to improve the model’s robustness to variations in neural activity. We trained all models for 200 epochs using Adam as the optimization algorithm and monitored the event rate loss metric. We split the dataset, obtained from refs. ^50,51^, into training and testing sets made up of 80% and 20% of the data in a five-fold cross-validation. In repeated ten-fold steps of cross-validation^60^, we split the data into 90% training sets and 10% testing sets. We trained the SNN model with training data while the testing set was only used to assess the final performance of the model in predicting the *x* cursor velocity. The predicted cursor velocity was compared with the original cursor velocity. As a measure of comparison, we calculated the correlation (*r*) between the two^58^. In preprocessing, we subtracted the mean of cursor velocity for better decoding performance.

In the ten-fold cross-validation, the data were repeatedly split into testing and training sets, such that the testing set in each fold comprised a different 10% of the total dataset. We independently trained the model three times for each condition and determined the correlation between the original x cursor velocity and the prediction of the model. To analyse the statistical significance, we performed Fisher *Z-*transformation on the correlation coefficient and conducted a paired *t*-test on the transformed values comparing the jittered spike and fixed spike conditions to determine if they yielded statistically different results.

## Supplementary information

Supplementary Information Supplementary Figs. 1–9, Notes 1–6 and Tables 1 and 2.

Supplementary Code Example data and code.

## Data Availability

The data that support the findings of this study are available from the corresponding author upon reasonable request.

## Code availability

Custom-developed MATLAB code for RF simulation and demodulation for the ASBIT protocol is available in the Supplementary Information and from the corresponding author upon reasonable request.
