# Supplementary material for: An asynchronous wireless network for capturing event-driven data from large populations of autonomous sensors
Source: Nat Electron. 2024 Mar 19;7(4):313–24. doi: 10.1038/s41928-024-01134-y (PMC11078753; doi:10.1038/s41928-024-01134-y)
Supplement: Supplementary file 1 — Supplementary Figs. 1–9, Notes 1–6 and Tables 1 and 2. [file 41928_2024_1134_MOESM1_ESM.pdf]

nature electronics

Article

https://doi.org/10.1038/s41928-024-01134-y

An asynchronous wireless network for capturing event-driven data from large populations of autonomous sensors

In the format provided by the authors and unedited

nature electronics

Article

https://doi.org/10.1038/s41928-024-01134-y

An asynchronous wireless network for capturing event-driven data from large populations of autonomous sensors

In the format provided by the authors and unedited

Table of contents

Supplementary Note 1. The choice of Gold code and matched filter synthesis

Supplementary Figure 1. Gold code generator schematic and circuit implementation

Supplementary Figure 2. Example of experimental I/Q data

Supplementary Figure 3. A synthesis process to generate matched filter waveforms

Supplementary Note 2. Analysis of Received Signal Strength Indicator (RSSI), signal-to-noise (SNR), and efficiency

Supplementary Figure 4. Relationship between wireless transfer efficiency, Received Signal Strength Indicator (RSSI) and signal-to-noise ratio (SNR)

Supplementary Note 3. Comparison between an ASIC with free running on-chip oscillator and a clock frequency divider approach

Supplementary Figure 5. Gold code generation and clock frequency stability in the experimentally measured ASBIT microchips

Supplementary Note 4. Fundamental network capacity and coding gain in the wireless ASBIT network

Supplementary Figure 6. Flow chart of the event recovery process at the receiver

Supplementary Note 5. The comparison between the ASBIT and other communication methods used in sensor devices specific to neural implants

Supplementary Table 1. Comparison with communication method in state-of-the-art microimplants

Supplementary Figure 7. The scheme for preprocessing open source multichannel primate electrophysiological neuronal spike data

Supplementary Figure 8. The spike error rate (SER) in transmitting 8,200 nodes of neural spike data

Supplementary Table 2. Correlation analysis between the original x-velocity of the cursor and its reconstructed value using a neural decoder

Supplementary Note 6. Power and loss considerations for building a wireless power transfer system for multisensory neural interface.

Supplementary Figure 9. Possible wireless energy transfer configuration for a large-scale neural interface

References

Supplementary Note 1. The choice of Gold code and matched filter synthesis

Gold codes are one type of pseudo-random (PN) sequence that are commonly used in spread-spectrum communication. A set of Gold codes provides quasi-orthogonality, which allows multiple users to share the same frequency band without being affected by mutual interference. The use of Gold codes helps to improve signal quality in noisy environments in general by allowing the receiver to filter out unwanted noise and interference. In the context of the ASBIT protocol, such noise and interference would be mainly due to background signals transmitted from other nodes. Furthermore, compared to other PN generators such as the Kasami code generator, the circuit implementation of the Gold code generator is simple, as shown in Supplementary Fig. 1a, resulting in a low-power, small footprint ASIC design [1]. However, the Gold code may not be the optimal choice for all circumstances, and other pseudorandom (PN) sequences could also be utilized in the ASBIT protocol of this paper.

In the ASBIT protocol, matched filters are important for event recovery by RF demodulation. To synthesize the matched filters, the pre-discovered digital Gold code at 10 Mbps is first converted to an analog waveform which matches with the backscattering waveforms from the specific target chip collected at the RF receiver. Supplementary Fig. 3 demonstrates the synthesis process of generating backscattering signals from the digital Gold code on RF microsensors. The method section ("Modeling of backscattering signals from RF microsensors for matched filter design") provides relevant theory and equations for the process. Briefly, the gold code sequence is first encoded into binary phase shift keying (BPSK) as a digital waveform using a 30 MHz digital clock. It is then transmitted through backscattering by toggling a capacitor in the transmission circuit on the microchip. The external RF receiver, a software-defined radio (SDR) in our case, collects the signal and down-converts the backscattered sideband (~945 MHz) to DC. This entire process can be modeled simply by down-converting the BPSK-encoded gold code signal from 30 MHz to DC which results in an estimated analog waveform of the Gold code. In the event recovery step of the ASBIT protocol, we used this analog Gold code waveform to generate a specific matched filter. Supplementary Figs. 1b and 1c show photos of the relationship and the size of the Gold code finite state machine (FSM) in relation to the overall wireless chip.

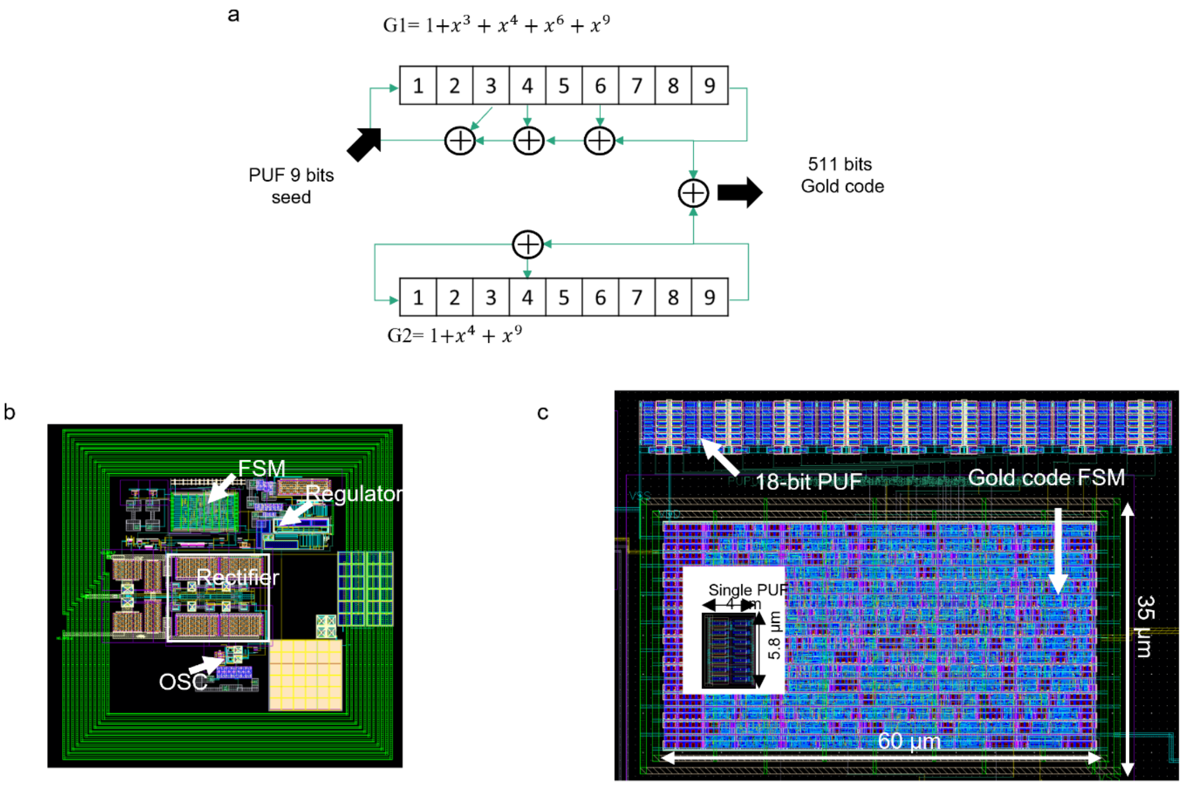

Supplementary Figure 1. Gold code generator schematic and circuit implementation. a) Schematic of a 511-bit Gold code generator, where a linear-feedback shift register is combined with a physically unclonable function to generate the Gold code sequence. b) The layout of the prototype wireless RFID microchip where the power harvesting rectifier and the perimeter circling on-chip coil occupy most of the circuit area whereas the footprint of the finite state machine (FSM) is considerably smaller. c) Magnified view of the Gold code FSM showing its footprint of 35 µm × 60 µm, together with the physically unclonable function (PUF) seed circuit which has a unit size of 4 µm × 5.8 µm. The PUF provides a random number unique to each microchip in order for the chip to synthesize its unique gold code. To generate a Gold code, we utilized 13 bits of the PUF as a seed sequence. This allowed us in principle to synthesize a total of 8191 bits of Gold code. However, to reduce mutual interference during communication, only a portion of the Gold code consisting of 511 bits was actually transmitted. By using a 13-bit seed sequence instead of a 9-bit seed sequence, we were able to minimize the likelihood of PUF-induced collisions (i.e., the very same PUF sequence was present in several chips).

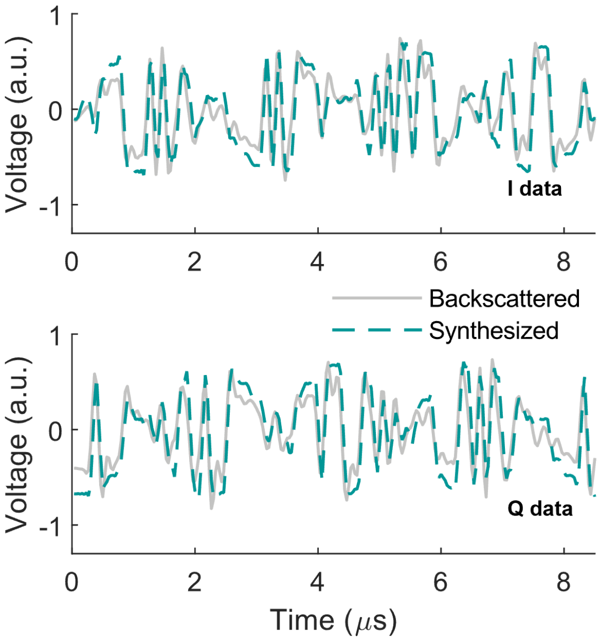

Supplementary Figure 2. Example of experimental I/Q data measured from the wireless chip (gray trace) compared with that synthesized by a matched filter as generated by converting a digital bit stream into analog I/Q waveform in the ASBIT network simulation model (green trace).

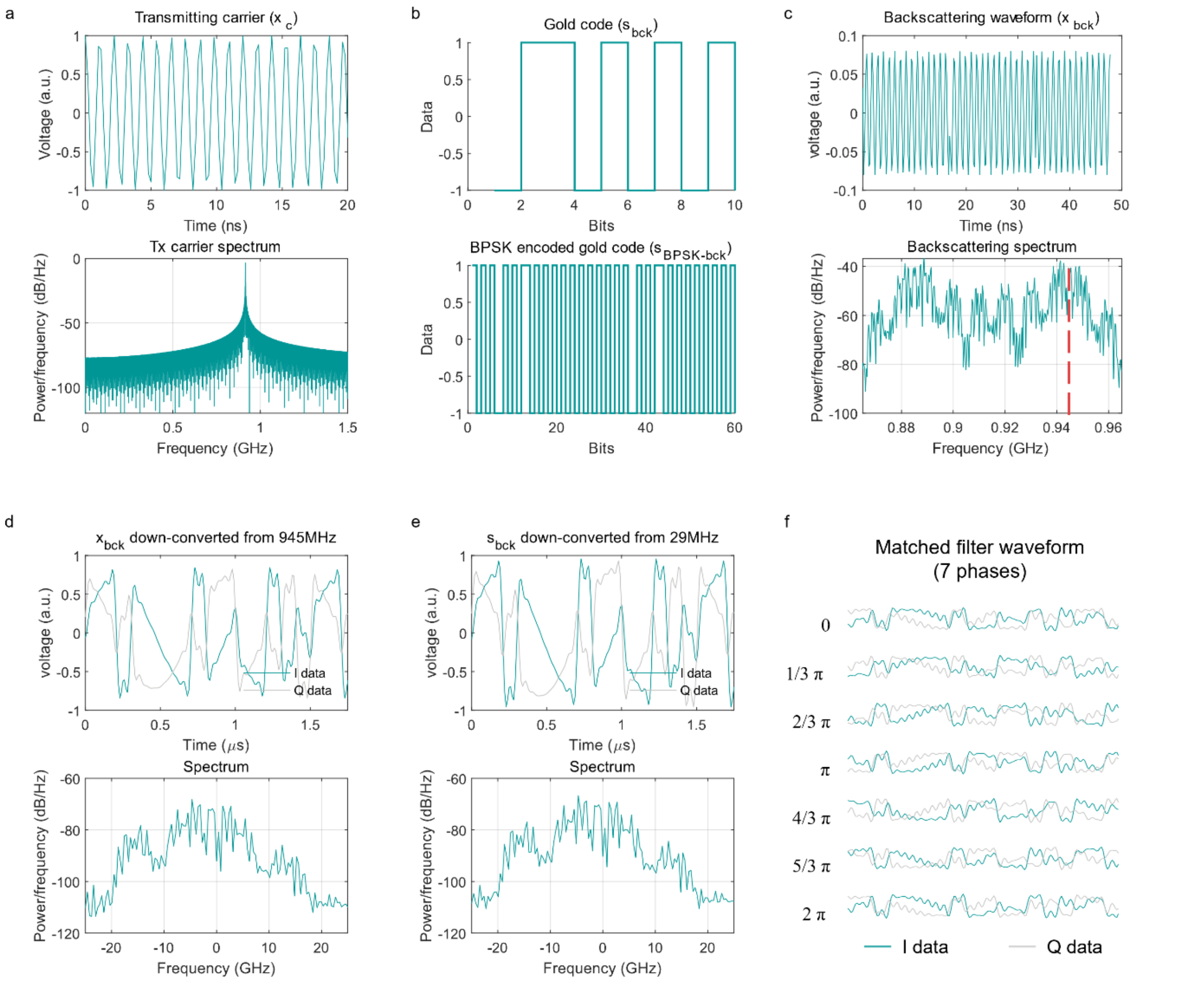

Supplementary Figure 3. A synthesis process to generate matched filter waveforms according to equations (1)-(6) in Methods ("Modeling of backscattering signals from RFID microsensors for matched filter design"). a) Downlink 915 MHz carrier wave (x_c) in Main Eq (1) (time resolution 200 ns) and its spectrum. b) Gold code sequence (s_{bck}) encoded by binary phase shift keying (BPSK) in the digital waveform $s_{BPSK\text{-}bck}$. c) Backscattered signals (x_{bck}) generated by modulating x_c by $s_{BPSK\text{-}bck}$, and the corresponding spectrum of x_{bck}. The red dotted vertical line shows the center frequency of the target side band (946 MHz; i.e. 915 MHz + clock of the target node at 31 MHz); d) I and Q data of x_{bck} waveforms down-converted from 945 MHz to DC displaying a 1 MHz residual clock frequency, i.e. the particular node clock frequency subtracted by the nominal clock frequency of 30 MHz; its finite spectrum reflecting the bandwidth of the bit sequence (10 Mbps). e) Waveforms of I and Q data and their spectra obtained by down-converting s_{bck} from 29 MHz with a residual clock frequency of 1 MHz. According to Main Eq. (5), x_{bck} waveforms down-converted from 945 MHz to DC should be equivalent to that of s_{bck} down-converted from 29 MHz to DC. f) Examples of matched filter waveforms showing phase dependent I and Q data with the residual clock of 3 MHz, which shows that the waveform is determined by the phase delay and the sampling phase (See Methods for detailed description).

Supplementary Note 2. Analysis of Received Signal Strength Indicator (RSSI), signal-to-noise (SNR), and efficiency

In the ASBIT backscattering method, the wireless transfer efficiency is crucial for the device's operation for both energy harvesting from the external transceiver (Tx) downlink while using it for backscattered communication (Supplementary Fig. 4a). The Tx transmits power (P_{tx}), which is captured by an on-chip coil (Rx) as P_{tx} + η (defined as the wireless efficiency at 915 MHz), where η is determined by the coupling factor between Tx and Rx coils (in case of near-field) or antenna gain (in case of far-field) plus the path loss. The received energy is then converted to a backscattering signal at a conversion efficiency η_c by the device circuits in the sensor microchip and transmitted back to the transceiver (Tx). The backscattering signal is then subject to wireless efficiency (η + c, where c is additional path loss at 945 MHz).

Thus, the level of the Received Signal Strength Indicator (RSSI) in the system can be expressed as:

$$RSSI\ (dBm) = P_{tx}\ (\text{dBm}) + 2\ \times\ \eta + \text{c}\ + \eta_c \qquad \text{(Supplementary Eq. 1)}$$

Using this equation, we measured the change in RSSI with varying Tx power (Supplementary Fig. 4b). The plot on the left shows the chip turning on at a Tx power of 13 dBm to achieve the target clock frequency. The plot on the right further shows how, when Tx power increased, the RSSI does not increase linearly, indicating that η_c is not constant and can vary with the Tx power in our backscattering modulation circuit. This design of circuit configuration alleviates the near-far problem, as the node with higher energy does not generate backscattering signals simply proportional to its power level.

Since the chips operate in fully wireless mode, we cannot precisely determine the conversion efficiency. Still, according to Supplementary Eq. 1, we can estimate the relationship between conversion efficiency and wireless transfer efficiency. Based on a combination of ASIC circuit and RF simulations, we estimated the threshold RF level for a microchip to be -16 dBm, 25.11 µW [2]. In that case, η can be solved as -29 dB so that the efficiency η_c + c can be in the range of -24 dB.

From another perspective and according to the definition of Signal-to-Noise Ratio (SNR), RSSI can be also solved as

$$SNR = RSSI\ (dBm) - Noise\ floor\ (dBm) \qquad \text{(Supplementary Eq. 2)}$$

Therefore, the wireless transfer efficiency (η) and its relationship to SNR can be determined using pre-discovered values of P_{tx} , η_c + c, and noise floor (dBm) for the specific case. In our microchips and from their RF interface, η_c + c was measured as -24 dB and P_{tx} of 24 dBm was chosen, since this is at the regulatory limit for the Specific Absorption Rate (SAR), 10 Wkg^{-1} SAR averaged over 10 g of the tissue [3]. Also, since we do not use any additional means to control the background noise (such as a Faraday cage), the noise floor of the system, measured by a software-defined ratio, was -75.75 dBm. At this noise level, the relationship between SNR and corresponding wireless transfer efficiency can be solved using Supplementary Eqs. 1 and 2. For example, with an SNR of 3 dB, the estimated RSSI is -72.75 dBm, and the predicted wireless power transfer efficiency is -36.375 dB. Therefore, SNR values used in plots Fig. 3e and Fig. 4c also provide an indirect indication of the wireless efficiency of the system. We note that the relationship between SNR and the wireless transfer efficiency can vary in other wireless systems based on factors such as coil size, conversion efficiency, and noise floor. Therefore, the analysis presented here is accurate only for our specific experimental setup.

We characterized wireless power harvesting in the fabricated chips using the 3-coil system shown in Fig. 2c. Supplementary Fig. 4c illustrates the measured and simulated efficiency based on the chip's location within the relay coil. We determined the efficiency of the wireless link by subtracting the required on-chip

energy (found to be -16 dBm in the circuit simulation) from the minimum Tx power needed to activate the chip. The measurements indicated an efficiency range of -35.45 dB to -26.95 dB, with the average measured efficiency 4.1 dB lower than the simulated efficiency on average. This discrepancy could be attributed to an impedance mismatch between the on-chip coil and circuits. Note that we have also made improvements in our current system by optimizing the on-chip coil design, resulting in a lower loss of only 4.1 dB compared to the 7 dB loss in our previously reported case [2].

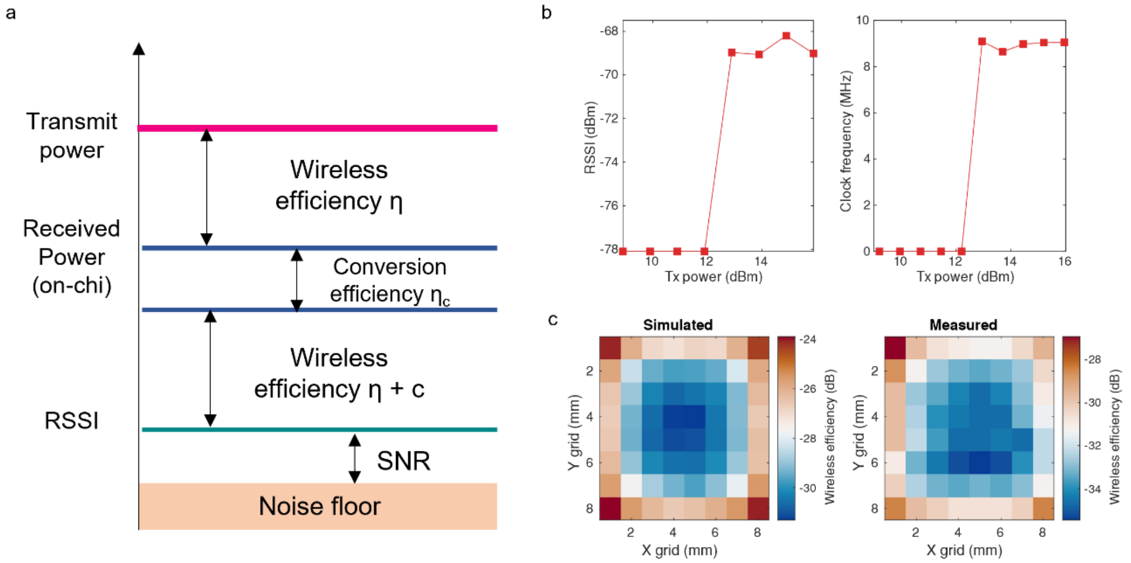

Supplementary Figure 4. Relationship between wireless transfer efficiency, Received Signal Strength Indicator (RSSI) and signal-to-noise ratio (SNR). a) Schematic diagram illustrating the levels of RSSI and SNR in our ASBIT backscattering scheme. The SNR level is influenced by various factors including wireless transfer efficiency (η @915 MHz), backscattering efficiency (η + c, @945 MHz), conversion efficiency (η_c), and the noise floor. b) Dependence of RSSI of the backscattering signals and clock frequency of the microchip on the Tx power; c) Comparison between simulated and measured wireless power transfer efficiency at 64 different locations within the relay coil perimeter, indicating a 4.1 dB difference due to possible impedance mismatch and circuit process variance.

Supplementary Note 3. Comparison between an ASIC with free running on-chip oscillator and a clock frequency divider approach

First, we assessed the consistency of the chips individually, ensuring their ability to generate the same Gold code pattern over a finite period for the ASBIT communication. All 78 chips with the free-running oscillator consistently produced backscattering signals every 20 ms. The left plot in Supplementary Figure 5a shows that, among these, 47 chips generated identical Gold codes across all 1,000 packets, and 18 chips transmitted the same code more than 90% of the time. We also tested the Gold code repeatability of 65 chips with the frequency divider replacing the on-chip oscillator as shown in the right plot of Supplementary Fig. 5a. Out of these, 40 chips backscattered the same Gold code over 1,000 times. In the experiments described in the Main text, we selected a target group of chips that consistently generated the same Gold code. All remaining chips were used to generate background signals for simulating a large-scale network. The instability of the PUF circuit, the seed for the Gold code, caused the Gold code synthesizer to generate variant codes. Such instability is well known in PUF which comes from measurement noise and environmental fluctuations [4]. Implementing a PUF error correction circuit, which samples PUF sequences repeatedly and finds the most frequent bits, can improve the yield since the PUF instability was temporary most of the time in this and our previous study [2].

Supplementary Fig. 5a also shows the variation in clock frequencies among the chips with the oscillator and the frequency divider. The clock frequency ranges from 10 to 11.17 MHz in our on-chip relaxation oscillator design, commonly chosen for its low power and area, that incorporates a capacitance subject to foundry process variance. We addressed this clock variance by designing a set of matched filters specific to each chip that could reliably detect the Gold code waveform. In the frequency divider-based ASBIT microchip, on the other hand, most chips generated a 9.53 MHz clock from a down-converted 28.59 MHz signal. Therefore, the chip-to-chip clock variance was negligible, except for a few outliers, which facilitated the demodulation step. Also, as shown in Supplementary Fig. 5b, the clock drift in the divider-based system was significantly lower compared to the oscillator-based system, leading to a consistent backscattered Gold code waveform. This accelerated the demodulation process as only three matched filters were required to account for sampling phase variance.

The cost of using the frequency divider approach requires more incoming RF energy compared to the oscillator-based system, leading to a tradeoff. As shown in Supplementary Fig. 5c, the average power required to turn on the divider-based microchip is approximately 5 dBm higher than that of the oscillator-based chips. This higher power requirement may be attributed to the higher input signal needed to cross the threshold voltage of the input differential amplifier, which may also explain the observed variance among chips. Nonetheless, we observed that some chips with dividers were able to operate with only a 2 dB increase in Tx power compared to those with on-chip free oscillators, indicating a potential for further improvement.

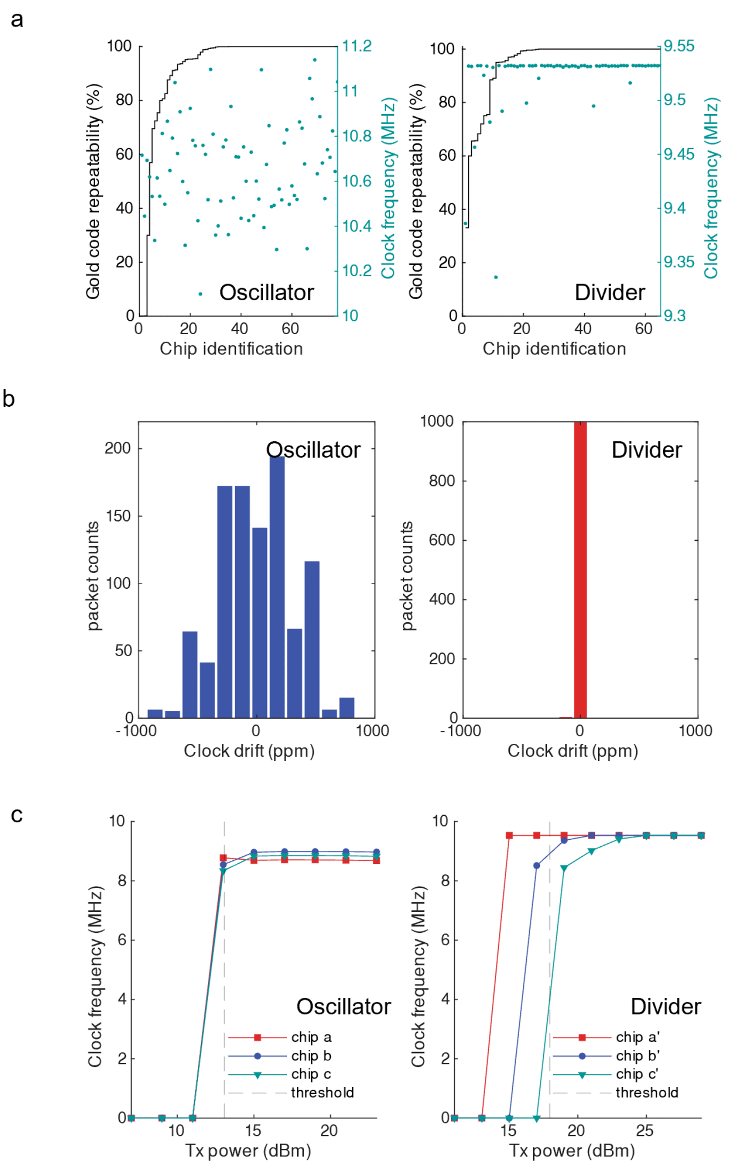

Supplementary Figure 5. Gold code generation and clock frequency stability in the experimentally measured ASBIT microchips. **a)** Left: Gold code packet repeatability and clock frequency variance among the chips of 78 microchips with free running oscillator clocks tested with 1,000 packets per chip. Right: Gold code repeatability and clock frequency variance of 65 microchips using the on-chip frequency divider tested with 1,000 packets each, showing the nominal clock frequency of 9.54 MHz in the majority of chips except for a few outliers. **b)** Histogram of the clock frequency drift of 1,000 packets from the oscillator and frequency divider, respectively. **c)** Clock frequency as a function of Tx power for three microchips randomly selected from a population of chips with free-running oscillator and frequency divider circuits, respectively, with the vertical gray dash line indicating the average threshold level for all available chips in each case. Abbreviation: ppm: parts per million.

Supplementary Note 4. Fundamental network capacity and coding gain in the wireless ASBIT network

While we evaluated the scalability of the ASBIT network using experimental data collected from microfabricated chips, one can also estimate the ideal network capacity for the proposed network. In the simplified limit of equal backscattered amplitude S from all nodes (i.e. ignoring the near-far problem), the SNR can be defined as follows, accounting for interference among all nodes [5-7]

$$SNR = S/[(N-1) \times S + \eta] \quad \text{(Supplementary Eq. 3)}$$

where N is the number of nodes in the network and η is the background noise from spurious interference plus thermal noise within the total spread bandwidth W. The ratio of energy per bit to noise power spectral density (E_b/N_0) can be expressed as

$$E_b/N_0 = \frac{W/R}{(N-1)+(\eta/S)} \quad \text{(Supplementary Eq. 4)}$$

while R denotes the bit data rate before spreading. Then, the network capacity in terms of the number of supported nodes becomes

$$N = 1 + \frac{W/R}{E_b/N_0} - \frac{\eta}{S} \quad \text{(Supplementary Eq. 5)}$$

Here, W/R is generally referred to as the CDMA processing gain, or coding gain, L_c. Given that $W \approx 1/T_c$ (T_c is the 'chip' data duration, the 'chip' defined as each bit in the Gold code) for a given predefined bit rate R, this equation implies that the maximum allowable number of nodes increases as the chip duration becomes shorter and the Gold code gets longer, which demands a wider bandwidth W. In our case, the bandwidth W is limited to 10 MHz by the present ASIC design and implementation constraints. Since the bit rate R is inversely proportional to the length of the Gold code, L_c and thus the network capacity is simply proportional to the length of the Gold code. Assuming an application acceptable event error rate (EER) of 10^{-3} for a path loss is 40 dB, and imposing a requirement for E_b/N_0 of 7 dB for 511-bit BPSK Gold code, we obtain that a single CDMA channel can accommodate 169 nodes in the ASBIT protocol with a thermal noise density of -174 dBm/Hz. Importantly, however, under the assumption of sparsity in the ASBIT scheme with only some 5% of band utilization per node (50 Hz event rate), the network capacity of the ASBIT protocol for the case of a 511-bit Gold code is estimated to be 3,380 nodes. In this ideal limit, one has ignored the impact of e.g. clock variance across nodes, clock drift at each node, and the amplitude variance (near-far problem) which reduces the limits the network capacity. Note, however, that in our paper, driven by experimental data on actual chips, we have taken into account a number of non-ideal factors to evaluate the practical network capacity of a functional ASBIT protocol while applying specialized techniques such as demodulation through discrete timing.

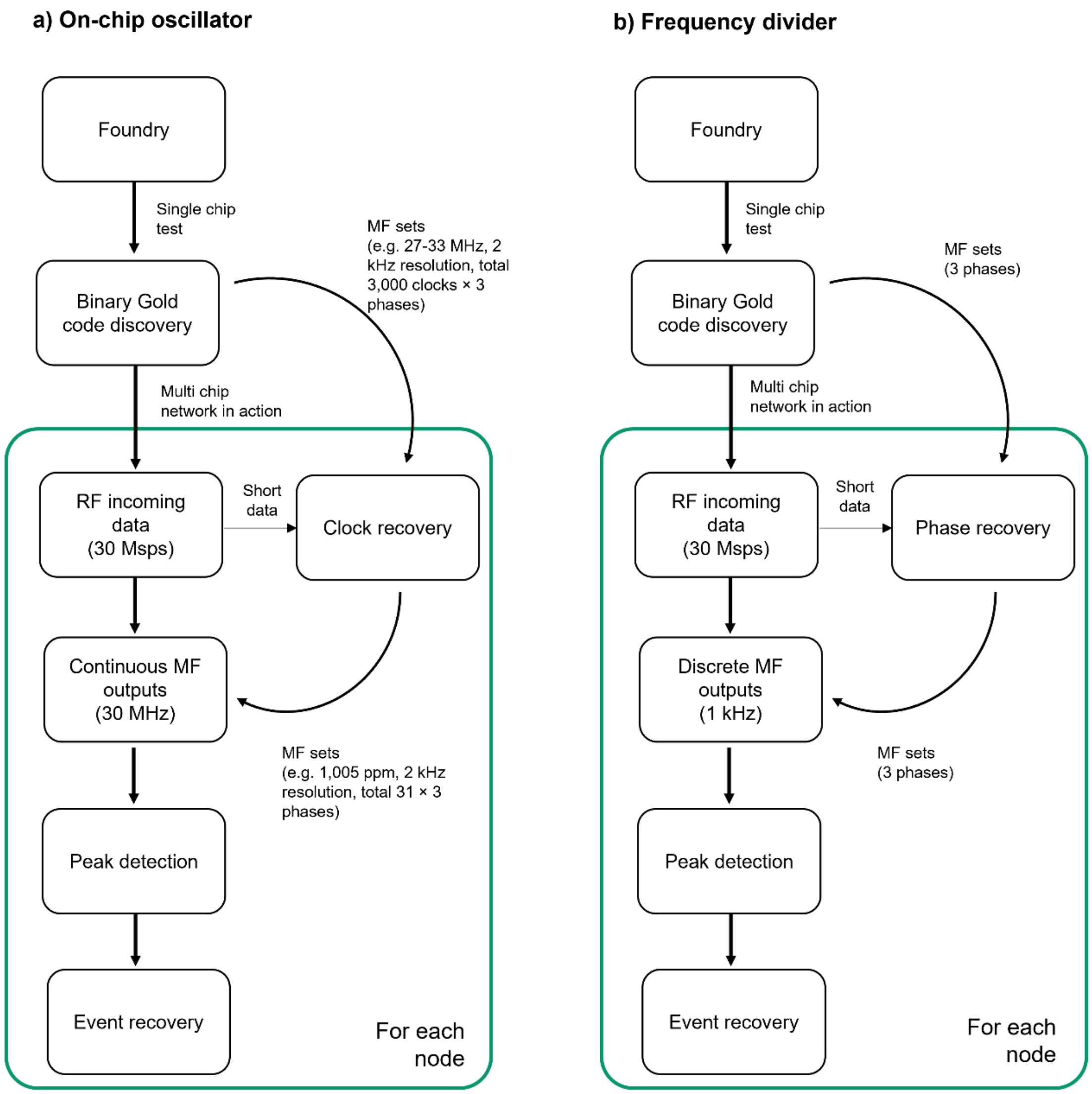

Supplementary Figure 6. Flow chart of the event recovery process at the receiver, i.e. demodulation of data from ensembles of RF microsensors encoded according to the ASBIT protocol; a) a free running oscillator as the on-chip system clock; b) Case of baseband carrier derived on-chip frequency divider for clock generation. Abbreviation: MF: matched filter.

Supplementary Note 5. The comparison between the ASBIT and other communication methods used in sensor devices specific to neural implants

The ASBIT protocol is distinct from other conventional communication methods, whether proposed or implemented for specific applications, such as for wireless neural implants, because it does not need to allocate specific resources like timeslots and frequency bands to nodes. Here, we first summarize the key points about the ASBIT protocol and then show examples from the neural implant space. We note that as the ASBIT protocol targets wireless RFID-type smart sensor networks, an emerging field still under exploration. In research on various random-access protocols on RFID tags (passive, no sensing capability), a sophisticated anti-collision protocol using time-division multiple access (TDMA) is proposed as one avenue [8-9] or other deterministic tree-search-based methods [10, 11] or probabilistic time-slotted methods [12-14]. Whether deterministic or probabilistic, their scalability is still limited due to penalties from time scheduling or packet collisions and the maximum number of sensor nodes remains well below fundamental limits. In our own recent work with RFID-type neural microsensors as brain implants, we proposed a TDMA method, leveraging a call-and-response type of bidirectional communication [2] yet found that the number of sensors would be limited to ≈770 incurring a system latency of 100 ms due to the spectral penalty imposed by the downlink.

The aggregate network capacity of the ASBIT system with frequency dividers has been estimated to be 100,000 events per second, with a single node able to report any number of events below the upper limit. To put this in perspective, the sensor in the ASBIT system can be thought of as a device transmitting 1 kbps rate of data, assuming a bin size of 1 msec and an event rate of 50 Hz (5% ones, backscattering, and 95% zeros, silence). This yields an aggregate data rate equivalent to 2 Mbps, which can increase to 10 Mbps if the event rate drops to 10 Hz. With an RF bandwidth of 10 MHz and BPSK encoding, the spectral efficiency of the ASBIT system is already approaching the theoretical maximum of 1 bit/Hz. By contrast, our own previous TDMA method [2] had a spectral efficiency of 0.77 bit/Hz even allowing for a 100 msec system latency.

Turning to RF communication systems used or proposed in implants (Table S1), the ASBIT system uses a much higher system clock for higher data rates, which can lead to higher current consumption. However, we reduce power consumption by utilizing sparse backscattering for data communication and optimizing circuits to improve impedance matching. Our microchip operates with 25 μW, a number which would increase by 3.2 μW when including the sensor circuit for neural recording which was not included in our current prototype ASBIT communication chip.

A broadband neural recording device described in [15] has a data rate of 800 kbps, while an 8-channel field potential (LFP) recording system presented in [16] has a data rate of 205 kbps. Both systems generate backscattering for communication using impulse radio ultra-wideband (IR-UWB) or load shift keying (LSK), which allocate timeslots or frequency bands entirely to a single node. However, these systems have much larger current consumption than in our case. The ‘neural dust’ system in [17] uses only passive transistors for backscattering, which reduces power consumption but limits the signal quality. And, especially, the scalability of the system beyond a handful of sensors appears quite limited. The optically powered ‘Mote’ interface described in [18] achieves a low power consumption of less than 1 μW by utilizing optical pulse position modulation for data communication. However, its performance has only been evaluated in limited conditions that do not account for losses in fully assembled optical-electrical devices the monolithic microfabrication of which poses a major heterogeneous integration challenge. By contrast, our device principle is fully compatible with a monolithic system-on-chip structure, with all circuit blocks fully integrated into a single piece of silicon. Microfabrication related issues and associated losses/yield are therefore absent in our case result. When applying this technology to wireless neural

interfaces, the only additional loss that needs to be accounted for is the transmission loss in biological tissue. These losses have been studied and are presented in Supplementary Fig. 9.

Table S1| Comparison with communication method in state-of-the-art microimplants

	[4]	[5]	[6]	[7]	Current work
Number of node(s)	1	1	1	Up to 1000	Up to 8000
Wireless Powering	131 MHz RF 3-coil	433 MHz RF 3-coil	1.85 MHz Ultrasonic	Optical	915 MHz RF 3-coil
External Tx power (mW)	-	-	0.12	-	Up to 316
Telemetry	Backscattering IR-UWB	Backscattering LSK	Backscattering	Pulse Position Modulation	Backscattering BPSK (ASBIT)
Uplink data rate (Mbps)	0.8 Mbps	0.205 Mbps	0.5 Mbps	0.6 -1.3 kbps (aggregate 0.6-1.3 Mbps)	1 kbps per node (Aggregate 1-10 Mbps)*
Technology	350 nm CMOS+ discrete	350 nm CMOS	Discrete	180 nm CMOS	65 nm CMOS
IC Area [mm^2]	1.1	12.25	0.032	0.0297	0.42
Energy harvester	Discrete coil	Discrete coil	Piezoelectric	Photovoltaic cell	On-chip Coil
Power supply (V)	1.8	1.5	-	1.5	0.8
Power Consumption (uW)	<300	92	<1	<1	<30

*** Determined by the sparsity of the target signal**

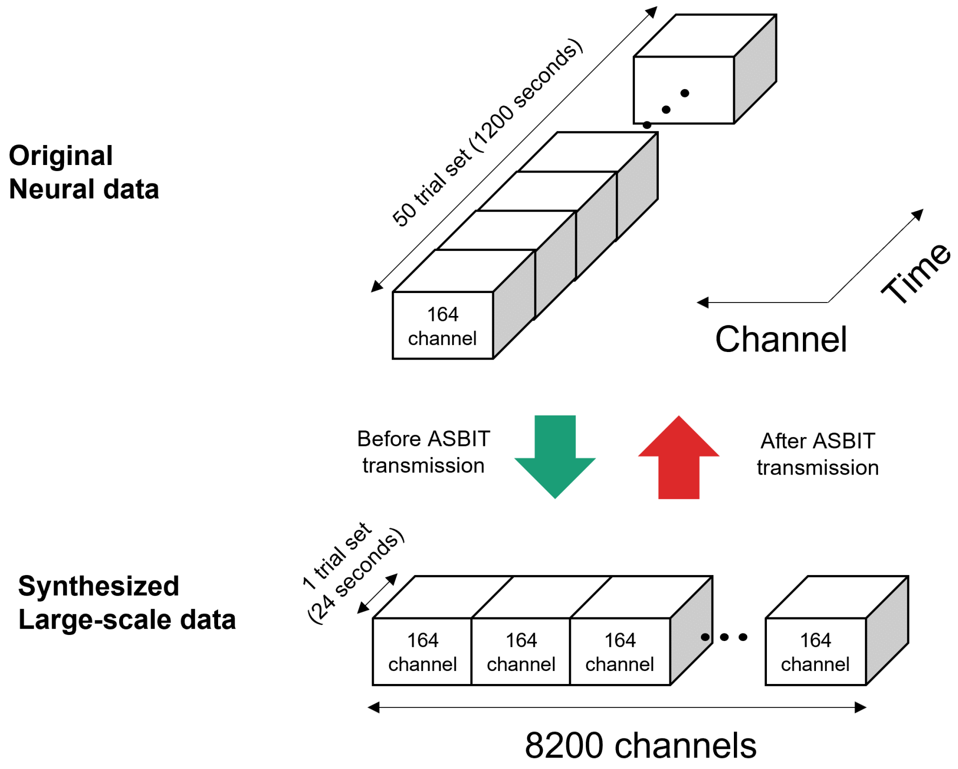

Supplementary Figure 7. The scheme for preprocessing open source multichannel primate electrophysiological neuronal spike data, recorded from the primate cortex by wired microelectrode arrays, here providing the input to an ensemble of wireless recording microchips using the ASBIT protocol. Available open source neural data was limited to only 164 spiking channels whereas the network capacity of the ASBIT protocol can reach up to several thousands. We increased the number of channels by transmitting neural data from 50 or 160 datasets simultaneously to generate an equivalent of 8,200 channels (164 neurons × 50 datasets) in the motor cortex (M1) and, 8,320 channels (52 neurons × 160 datasets) in the somatosensory cortex (S1). Following the ASBIT transmission step from the corresponding number of microsensors, we reconstituted and mapped the data back into the original number of channels for the use of this data to train a neural network model for brain-machine interface (BMI)-relevant neural decoding. All experimental neural data were obtained from [19, 20].

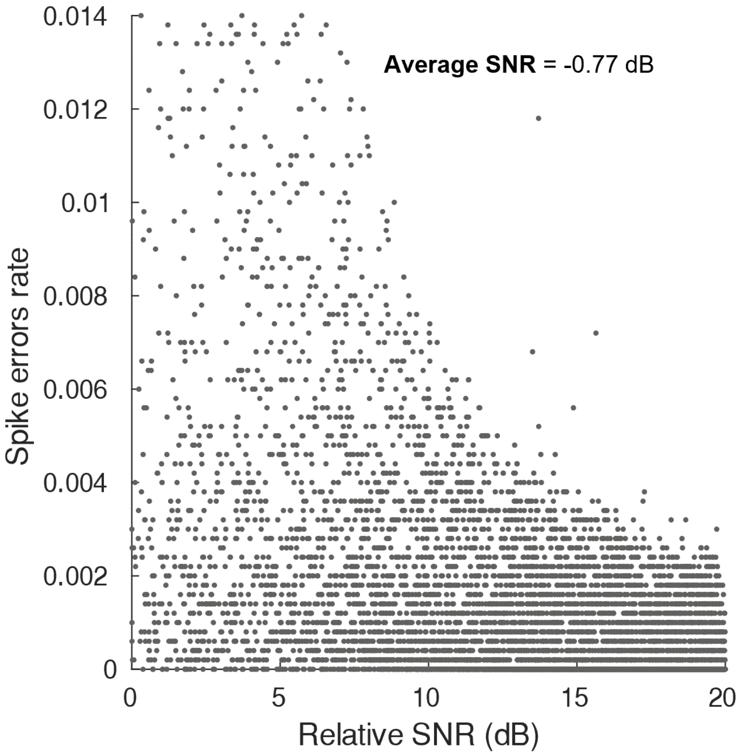

Supplementary Figure 8. The spike error rate (SER) in transmitting 8,200 nodes of neural spike data. The simulation addresses the near-far problem by varying the amplitude of backscattering which leads to differences in relative SNR (0 to 20 dB) among chips. The results indicate that nodes transmitting weaker signals, those located farther away from the external receiver, exhibit higher spike errors compared to those generating stronger backscattering as they are located closer to the external receiver.

Table S2 | Correlation analysis between the original x-velocity of the cursor and its reconstructed value using a neural decoder					
**Content**	**Correlation (5-folds)**				
M1 (collected in a wired system)	0.93911				
M1 (transmitted through the ASBIT protocol, SNR= -12.77)	0.658621	0.720138	0.752318	0.732099	0.717825
M1 (transmitted through the ASBIT protocol, SNR= -8.77)	0.794375	0.854243	0.874156	0.804641	0.843862
M1 (transmitted through the ASBIT protocol, SNR= -4.77)	0.878084	0.900917	0.905043	0.875753	0.89279
M1 (transmitted through the ASBIT protocol, SNR= -0.77)	0.913785	0.924004	0.929386	0.903682	0.921177
M1 (transmitted through the ASBIT protocol, SNR= 3.23)	0.926512	0.936714	0.942347	0.923157	0.935254
M1 (transmitted through the ASBIT protocol, SNR= 19.23)	0.922885	0.936211	0.943901	0.922071	0.936769
S1 (collected in a wired system)					
S1 (transmitted through the ASBIT protocol, SNR= -12.77)	0.671793	0.690964	0.671976	0.643869	0.58587
S1 (transmitted through the ASBIT protocol, SNR= -8.77)	0.808395	0.809847	0.808138	0.799064	0.788678
S1 (transmitted through the ASBIT protocol, SNR= -4.77)	0.874804	0.873204	0.873599	0.863752	0.859218
S1 (transmitted through the ASBIT protocol, SNR= -0.77)	0.903724	0.902957	0.910874	0.900415	0.897612
S1 (transmitted through the ASBIT protocol, SNR= 3.23)	0.922929	0.920866	0.924606	0.923442	0.913239
S1 (transmitted through the ASBIT protocol, SNR= 19.23)	0.928275	0.932218	0.933293	0.932113	0.922434

Supplementary Note 6. Power and loss considerations for building a wireless power transfer system for multisensory neural interface.

Although our work mainly demonstrates the communication idea in the ASBIT method, it is important also to anticipate possible limitations in wireless power transfer efficiency in building wireless neural or other body-implanted sensor networks. As explained in Supplementary Note 2, our present communication microchip requires a minimum power of 25.11 μW (-16 dBm), and a wireless transfer efficiency of -40 dB is necessary for chip operation at 24 dBm transmitting power. Although power consumption can be improved through smaller nodes or better ASIC circuit designs, and is not close to fundamental limits, we used these parameters to assess the feasibility of a wireless neural sensor interfaces with thousands of distributed devices.

As an example of a possible configuration, we simulated a 4-coil wireless link, including our previously reported approach [2] now with a 300 μm × 300 μm square microcoil in the perimetry of each sensor. The first relay coil was assumed to be placed 5 mm away from the Tx external transmitting coil (2 mm of skin and 3 mm of fat layers), while the second relay coil and the on-chip microcoil were coplanar and located 7 mm apart from the first relay coil (7 mm of the skull), as illustrated in Supplementary Fig. 9a. For epicortical applications, we assumed that electrodes attached to individual chips penetrate the cortex to access neural spikes. We then used Ansys, HFSS (high-frequency structure simulator) to solve for the quality factor of the coils and the coupling factor between them, and calculated the wireless transfer efficiency in the 4-coil system as described in [21]. The geometry of the Tx and microcoil on-chip, as well as the simulated efficiency in 2,025 locations spaced 300 μm apart within one quadrant of the transmitting coil, is shown in Supplementary Figure 9. (As a practical matter, the relay coils, deposited on flexible polymer substrates a fraction of a millimeter in thickness, are relatively simple to insert into tissue and have been used by us in rodent implants [2]).

Based on our simulations, we found that the wireless transfer efficiency between our the Tx coil and on-chip Rx microcoils ranged from -37.3 dB to -29.3 dB, resulting in power received on the chips ranging from 46.7 μW to 295.1 μW. All locations within one quadrant of the transmitting coil had an efficiency above -40 dB and each quadrant of the relay coil covers an area that can accommodate up to 900 microchips. With four symmetrical quadrants, up to 3,600 microchips can be placed within the Tx coil in this design, allowing for the simultaneous collection of spike signals from thousands of chips. However, the impedance mismatch between the coil and circuit and loading effects from other microchips can decrease the wireless transfer efficiency. Therefore, the current circuit design might require more than allotted 24 dBm of total Tx power, or a sophisticated resonance tuning mechanism to minimize the loading effects. Another path is to pursue the development of wireless sensors with lower power requirements than the -16 dBm number above, leveraging semiconductor technology at an advanced process beyond the 65 nm CMOS node. To achieve an 8,000 neural sensor network, as simulated in Fig.4 of the main text, additional choices can involve the use of multiple Tx external antennas [2] to distribute incident RF power across a wider area while remaining below SAR limits.

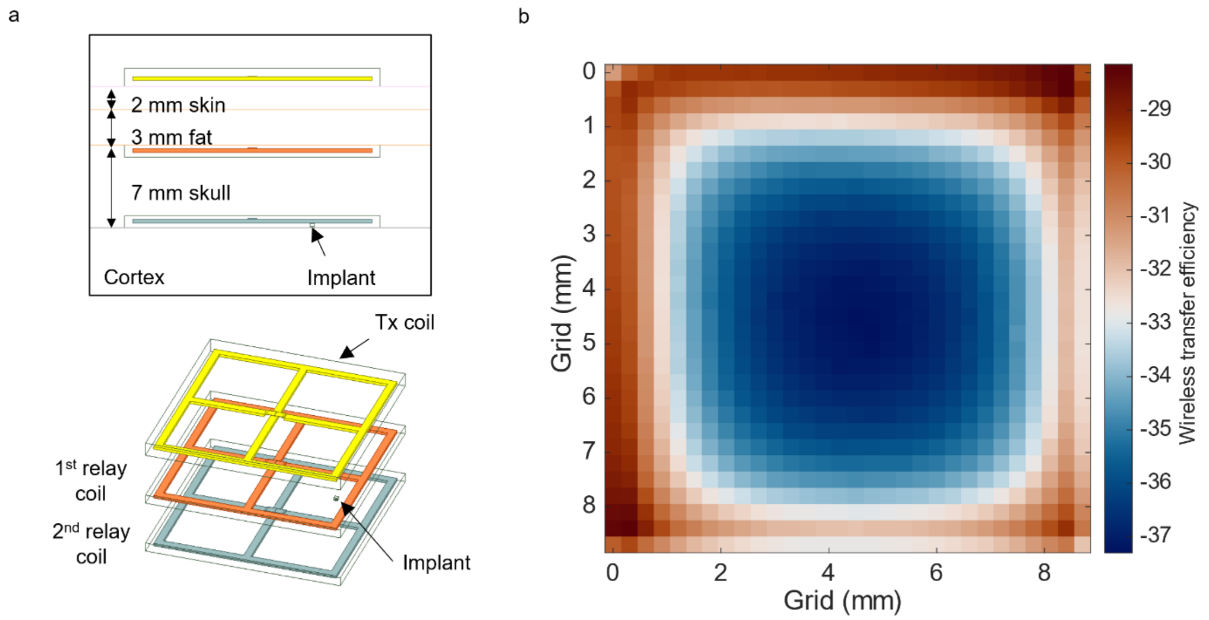

Supplementary Figure 9. Possible wireless energy transfer configuration for a large-scale neural interface. a) Schematic cross-section of a 4-coil system geometry for a wireless neural interface composed from several thousand microsensors. The system includes one external Tx coil (20 mm × 20 mm), two implanted relay coils (20 mm × 20 mm), and an on-chip microcoil on each implanted sensor chip (300 µm × 300 µm in area). The Tx coil is positioned over the skin, while the first and second sub-mm thick relay coils are inserted above and below the skull, respectively. Further details on the suggested coil design can be found in [2]. b) Heatmap of the simulated wireless transfer efficiency across the population of sensor chips for one quadrant of the 'window coils'. The results for the other three quadrants are symmetrical, and therefore only one quadrant is shown in the image. This plot presents that the wireless power transfer efficiency ranges from -37.3 dB to -29.3 dB in this 4-coil system. Each quadrant of the relay coil can accommodate up to 900 closely spaced microchips.

References:

[1] Kumar, V. Anil, Abhijit Mitra, and SR Mahadeva Prasanna. "On the effectivity of different pseudo-noise and orthogonal sequences for speech encryption from correlation properties." International Journal of Electronics and Communication Engineering 2.12 (2008): 2844-2851

[2] Lee, J. *et al*. Neural recording and stimulation using wireless networks of microimplants. *Nature Electronics* **4**, 604–614 (2021).

[3] Determining the peak spatial-average specific absorption rate (SAR) in the human body from wireless communications devices, 30 MHz to 6 GHz - part 3: Specific requirements for using the finite difference time domain (FDTD) method for SAR calculations of mobile phones. IEC/IEEE 62704-3:2017 1–76 (2017).

[4] Cao, Y., Zhang, L., Chang, C.-H. & Chen, S. A low-power hybrid ro PUF with improved thermal stability for lightweight applications. *IEEE Transactions on computer-aided design of integrated circuits and systems* **34**, 1143–1147 (2015).

[5] Proakis, J. G., Salehi, M., Zhou, N. & Li, X. *Communication systems engineering*, vol. 2 (Prentice Hall New Jersey, 1994).

[6] Gilhousen, K. S. *et al*. On the capacity of a cellular CDMA system. *IEEE transactions on vehicular technology* **40**, 303–312 (1991).

[7] Turkmani, A. M. & Goni, U. Performance evaluation of maximal-length, Gold and Kasami codes as spreading sequences in CDMA systems. In *Proceedings of 2nd IEEE International Conference on Universal Personal Communications*, vol. 2, 970–974 (IEEE, 1993).

[8] Klair, D. K., Chin, K.-W. & Raad, R. A survey and tutorial of RFID anti-collision protocols. *IEEE Communications surveys & tutorials* **12**, 400–421 (2010).

[9] Eom, J.-B., Yim, S.-B. & Lee, T.-J. An efficient reader anticollision algorithm in dense RFID networks with mobile RFID readers. *IEEE Transactions on industrial electronics* **56**, 2326–2336 (2009).

[10] Hush, D. R. & Wood, C. Analysis of tree algorithms for RFID arbitration. In *Proceedings. 1998 IEEE International Symposium on Information Theory (Cat. No. 98CH36252)*, 107 (IEEE, 1998).

[11] Myung, J., Lee, W. & Srivastava, J. Adaptive binary splitting for efficient RFID tag anti-collision. *IEEE communications letters* **10**, 144–146 (2006).

[12] Park, J., Chung, M. Y. & Lee, T.-J. Identification of RFID tags in framed-slotted ALOHA with robust estimation and binary selection. *IEEE Communications Letters* **11**, 452–454 (2007).

[13] Liva, G. Graph-based analysis and optimization of contention resolution diversity slotted ALOHA. *IEEE Transactions on Communications* **59**, 477– 487 (2010).

[14] Eom, J.-B. & Lee, T.-J. Accurate tag estimation for dynamic framed-slotted ALOHA in RFID systems. *IEEE Communications Letters* **14**, 60–62 (2009).

[15] Yeon, P., Bakir, M. S. & Ghovanloo, M. Towards a 1.1 mm2 free-floating wireless implantable neural recording SoC. In 2018 IEEE Custom Integrated Circuits Conference (CICC), 1–4 (IEEE, 2018).

[16] Ahmadi, N. et al. Towards a distributed, chronically-implantable neural interface. In 2019 9th International IEEE/EMBS Conference on Neural Engineering (NER), 719–724 (IEEE, 2019).

[17] Seo, D., Carmena, J. M., Rabaey, J. M., Alon, E. & Maharbiz, M. M. Neural dust: An ultrasonic, low power solution for chronic brain-machine interfaces. arXiv preprint arXiv:1307.2196 (2013).

[18] Costello, Joseph T., et al. "A low-power communication scheme for wireless, 1000 channel brain–machine interfaces." Journal of Neural Engineering 19.3 (2022): 036037.

[19] Glaser, J. I. *et al*. Machine learning for neural decoding. *Eneuro* **7** (2020).

[20] Benjamin, A. S. *et al*. Modern machine learning as a benchmark for fitting neural responses. *Frontiers in computational neuroscience* **56** (2018).

[21] RamRakhyani, Anil Kumar, Shahriar Mirabbasi, and Mu Chiao. "Design and optimization of resonance-based efficient wireless power delivery systems for biomedical implants." IEEE transactions on biomedical circuits and systems 5.1 (2010): 48-63.
